# The impact of body mass index on breast cancer incidence among women at increased risk: an observational study from the International Breast Intervention Studies

**DOI:** 10.1007/s10549-021-06141-7

**Published:** 2021-03-03

**Authors:** Samuel G. Smith, Ivana Sestak, Michelle. A. Morris, Michelle Harvie, Anthony Howell, John Forbes, Jack Cuzick

**Affiliations:** 1 Leeds Institute of Health Science, University of Leeds, Clarendon Way, Leeds, LS2 9NL UK; 2 Centre for Cancer Prevention, Wolfson Institute of Preventive Medicine, Queen Mary University of London, London, UK; 3 Leeds Institute for Data Analytics, School of Medicine, University of Leeds, Leeds, UK; 4 Prevent Breast Cancer Unit, Nightingale Breast Screening Centre, Manchester University NHS Foundation Trust, Manchester, UK; 5 Institute of Cancer Sciences, University of Manchester, Manchester, UK; 6 Breast Cancer Trials, Newcastle, Australia

**Keywords:** Weight, Breast cancer, Chemoprevention, Body mass index, Tamoxifen, Anastrozole

## Abstract

**Background:**

We investigated the association between body mass index (BMI) and breast cancer risk in women at increased risk of breast cancer receiving tamoxifen or anastrozole compared with placebo using data from the International Breast Cancer Intervention Studies [IBIS-I (tamoxifen) and IBIS-II (anastrozole)].

**Methods:**

Baseline BMI was calculated from nurse assessed height and weight measurements for premenopausal (*n* = 3138) and postmenopausal (*n* = 3731) women in IBIS-I and postmenopausal women in IBIS-II (*n* = 3787). The primary endpoint was any breast cancer event (invasive and ductal carcinoma in situ). We used Cox proportional hazards regression to calculate hazard ratios (HRs) for risk after adjustment for covariates.

**Results:**

There were 582 (IBIS-I) and 248 (IBIS-II) breast cancer events [median follow-up = 16.2 years (IQR 14.4–17.7) and 10.9 years (IQR 8.8–13.0), respectively]. In adjusted analysis, women with a higher BMI had an increased breast cancer risk in both IBIS-I [HR = 1.06 per 5 kg/m^2^ (0.99–1.15), *p* = 0.114] and in IBIS-II [HR per 5 kg/m^2^ = 1.21 (1.09–1.35), *p* < 0.001]. In IBIS-I, the association between BMI and breast cancer risk was positive in postmenopausal women [adjusted HR per 5 kg/m^2^ = 1.14 (1.03–1.26), *p* = 0.01] but not premenopausal women [adjusted HR per 5 kg/m^2^ = 0.97 (0.86–1.09), *p* = 0.628]. There was no interaction between BMI and treatment group for breast cancer risk in either IBIS-I (*p* = 0.62) or IBIS-II (*p* = 0.55).

**Conclusions:**

Higher BMI is associated with greater breast cancer risk in postmenopausal women at increased risk of the disease, but no effect was observed in premenopausal women. The lack of interaction between BMI and treatment group on breast cancer risk suggests women are likely to experience benefit from preventive therapy regardless of their BMI.

*Trial registration* Both trials were registered [IBIS-I: ISRCTN91879928 on 24/02/2006, retrospectively registered (http://www.isrctn.com/ISRCTN91879928); IBIS-II: ISRCTN31488319 on 07/01/2005, retrospectively registered (http://www.isrctn.com/ISRCTN31488319)]

**Supplementary Information:**

The online version contains supplementary material available at 10.1007/s10549-021-06141-7.

## Introduction

Breast cancer is the most common cancer in women worldwide and is the leading cause of cancer-related death [1]. Women with first- and second-degree relatives with breast cancer are at increased risk, particularly if multiple relatives are affected at a younger age [2]. Excess adiposity and weight gain are associated with an increased risk of breast cancer developing after the menopause but appear to reduce the risk of developing premenopausal breast cancer [3–6], particularly among those aged under 35 years [7, 8]. The association between weight and breast cancer risk is generally stronger for oestrogen-receptor-positive (ER+) breast cancers [9–11], although there is evidence for an association with ER-tumours [12].

Most evidence on the association between weight and breast cancer risk is from studies of women at general population risk, with adjustment for family history [6]. Observational data suggest that the relationship between BMI and breast cancer risk may be similar among women with and without a family history of the disease [13, 14]. However, data from the National Surgical Adjuvant Breast and Bowel Project (NSABP)-P1 preventive therapy trial are not consistent with this observation [15]. Among women assessed as having a 5-year breast cancer risk of at least 1.66%, higher BMI was associated with an increased risk of premenopausal breast cancer but not postmenopausal breast cancer. Additional evidence examining the relationship between weight and breast cancer risk in women at increased risk is needed to clarify this issue.

In the NSABP-P1 and P2 trials, the interaction between BMI and treatment group on breast cancer risk was examined [15]. Women in the P1 trial were randomised to receive either tamoxifen or placebo [16], and participants in the P-2 trial were randomised to receive either tamoxifen or raloxifene [17]. Analysis of both trials demonstrated no interaction between treatment group and BMI on breast cancer risk for either premenopausal or postmenopausal women. Similar observations have been made in the adjuvant setting with women using tamoxifen to prevent recurrence [18, 19]. However, higher BMI has been found to reduce the efficacy of aromatase inhibitors (AIs) such as anastrozole and letrozole in the adjuvant setting [20–24]. No data have been reported investigating the interaction between BMI and AIs on breast cancer risk in the primary prevention setting.

We used data from two large preventive therapy trials evaluating tamoxifen vs. placebo (International Breast cancer Intervention Study (IBIS-I) [25] and anastrozole vs. placebo (IBIS-II) [26] to estimate the relationship between BMI and breast cancer among women at increased risk of developing the disease.

## Materials and methods

### Participants and procedures

Data were from the IBIS-I and II trials, and patient characteristics and eligibility criteria have been reported previously [25–27]. Briefly, between April 1992 and March 2001. women in IBIS-I were recruited from centres in the UK, Australia, New Zealand and Europe if they were aged 35–70 years and had an increased breast cancer risk. Menopausal status was recorded at study entry, with postmenopausal defined if they had 12 consecutive months of amenorrhea or had an oophorectomy. Eligibility criteria were designed so that women had a relative risk of at least ten times higher than the general population if they were 35–39 years of age, four times higher if they were 40–44 years of age and two times higher if they were 45–70 years of age. In general terms, increased risk was determined from family history, previous lobular carcinoma in situ or atypical hyperplasia, by the Tyrer-Cuzick risk assessment tool [28].

IBIS-II participants were recruited between February 2003 and January 2012 and were included if they were postmenopausal, 40–70 years of age, and at increased risk of breast cancer due to their family history or a personal history of abnormal benign breast disease (atypical hyperplasia or lobular carcinoma in situ). Eligibility criteria in IBIS-II meant that women had a relative risk of breast cancer that was at least two times higher than the general population if they were aged 45–60 years of age, 1.5 times higher if they were aged 60–70 years, and four times higher if they were aged 40–44 years of age. Women not meeting the aforementioned eligibility criteria were eligible if they had a 10-year breast cancer risk of at least 5% as assessed by the Tyrer-Cuzick risk assessment tool [28]. Specific eligibility criteria for both trials are reported in the study protocol (Online Appendix).

IBIS-I participants were randomly assigned to 5 years of treatment with tamoxifen (20 mg) or matching placebo. The same approach was taken in the IBIS-II prevention trial comparing anastrozole (1 mg) with matching placebo. Women were actively followed for at least 5 years in both trials. In IBIS-I, women were followed up in the clinic or by telephone at 6-month intervals. In IBIS-II, women were followed up in the clinic at baseline, 6 and 12 months, and then annually until the 5-year endpoint. In addition to clinical visits, cancer events and deaths were reported to the trial office via national registries and postal questionnaires, and this has continued following completion of active treatment. All participants from both trials are being followed up on a regular basis unless they have died or withdrew their consent for long-term follow-up. Sites were contacted by the trial office for further information on each breast cancer. Women who had not developed breast cancer after a minimum of 10 years’ follow-up in IBIS-I were invited to enrol into the IBIS-II trial. Local ethics committees for each participating site approved the IBIS-I trial, and the UK North West Multicentre Research Ethics Committee approved the IBIS-II trial. Both trials were done in accordance with the Declaration of Helsinki, under the principles of good clinical practice. Both trials were registered (IBIS-I: ISRCTN91879928; IBIS-II: ISRCTN31488319).

#### Body mass index

In both trials, height (cm) and weight (kg) were measured by clinical staff at baseline, although there was no standard protocol for this process. BMI was calculated using these data, and women were classified as underweight or healthy weight (BMI < 24.9 kg/m^2^), overweight (BMI between 25.0 and 29.9 kg/m^2^) or obese (BMI ≥ 30.0 kg/m^2^). For continuous BMI, hazard ratios are presented for a 5 kg/m^2^ change.

### Statistical analysis

All analyses were completed on women for whom baseline BMI was available. The primary outcome was any breast cancer (invasive and ductal carcinoma in situ), and time to occurrence was defined as time from randomisation to diagnosis of breast cancer. Hazard ratios (HR) per 5-unit increase in BMI and corresponding 95% confidence intervals (CIs) were estimated by the proportional hazards regression model, both with and without adjustment for key covariates including age, hormone replacement therapy (HRT) use, current or previous history of smoking and baseline menopausal status (for IBIS-I only). In IBIS-I, we adjusted for current or previous use of HRT, whereas in IBIS-II, it was previous HRT use only. Additional covariates were also considered including age at menarche, age at menopause, oophorectomy and the Tyrer-Cuzick risk score [28]. However, there were no significant interactions between these factors and BMI on breast cancer risk, and therefore, they were not included in the models (data not shown). We also investigated interactions between treatment group and BMI on breast cancer risk. These analyses were based on likelihood ratio tests for an added interaction term. Time-to-occurrence curves were produced using the Kaplan–Meier method. All P values are two sided. Statistical significance was set at *p* = 0.05. Calculations were performed using STATA (version 13.1; Stata Corp, College Station, TX).

## Results

**Fig. 1:**
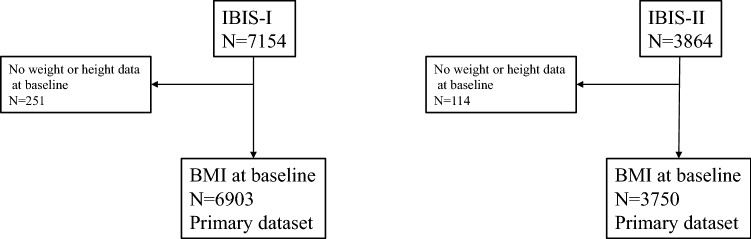
CONSORT flow diagrams for the IBIS-I and IBIS-II trials

Baseline BMI data were available for 6903 women in IBIS-I (3.5%, *n* = 251 missing) and 3750 women in IBIS-II (2.9%, *n* = 114 missing) (Fig. 1). There were 582 (IBIS-I) and 248 (IBIS-II) breast cancer events with a median follow-up of 16.2 years (IQR 14.4–17.7) and 10.9 years (IQR 8.8–13.0), respectively. Mean (SD) baseline BMI was 26.9 kg/m^2^ (5.2) in IBIS-I and 28.3 kg/m^2^ (5.4) in IBIS-II. In IBIS-I mean (SD) BMI was 26.8 kg/m^2^ (5.4) among premenopausal women and 27.1 kg/m^2^ (5.1) in postmenopausal women. Menopausal status was missing for 34 women in IBIS-I. Approximately one fifth (17%) of IBIS-I participants who had not developed breast cancer after 10 years of follow-up chose to enrol into the IBIS-II trial. Characteristics for IBIS-I and IBIS-II participants by baseline BMI category are shown in Table 1.

**Table 1:** Baseline characteristics by body mass index group (kg/m^2^) in the IBIS-I and IBIS-II prevention trials

	IBIS-I (tamoxifen vs. placebo) (*N* = 6903)			IBIS-II (anastrozole vs. placebo) (*N* = 3750)		
	BMI < 25 (*N* = 2878, 41.7%)	BMI: 25–29.9 (*N* = 2391, 34.6%)	BMI > 30 (*N* = 1634, 23.7%)	BMI < 25 (*N* = 1140, 30.4%)	BMI: 25–29.9 (*N* = 1420, 37.9%)	BMI > 30 (*N* = 1190, 31.7%)
Randomised to preventive therapy (%)	50.3	49.0	50.7	50.2	48.9	50.1
HRT use^a^ (%)	26.4	26.7	24.0	46.3	48.4	46.7
Current/history of smoking (%)	20.7	18.2	14.5	14.9	9.9	8.9
Postmenopausal (%)	50.5	57.9	54.9	100	100	100
10-year Tyrer-Cuzick % risk, median (IQR)^b^	5.5 (4.4–7.0)	5.8 (4.7–7.5)	6.0 (4.7–7.7)	7.0 (5.3–9.2)	8.0 (6.1–10.2)	8.0 (6.0–10.5)
Age (years), median (IQR)	49 (45–53)	50 (46–56)	49 (45–53)	59 (54–63)	60 (56–64)	60 (55–63)

*BMI* kg/m^2^, *IQR* interquartile range, *HRT* hormone replacement therapy

^a^In IBIS-I, HRT was either current or previous use, whereas in IBIS-II, it was previous HRT use only

^b^Missing IBIS-I: *N* = 221; IBIS-II *N* = 73

In unadjusted analyses, baseline BMI was associated with a significant increased risk of breast cancer among the IBIS-I cohort [HR = 1.07 per 5 kg/m^2^ (1.00–1.16), *p* = 0.049] and a significant increased risk in the IBIS-II cohort [HR = 1.21 per 5 kg/m^2^ (1.09–1.34), p < 0.001]. These estimates were similar after adjustment, although the association in IBIS-I no longer reached statistical significance [IBIS-I: HR = 1.06 per 5 kg/m^2^ (0.99–1.15), *p* = 0.114; IBIS-II: HR per 5 kg/m^2^ = 1.20 (1.09–1.35), *p* < 0.001].

**Fig. 2:**
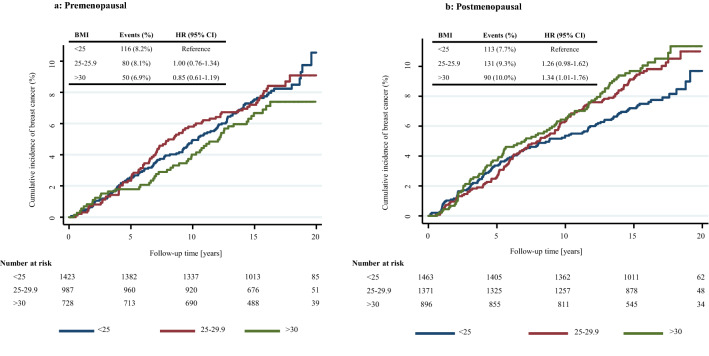
Kaplan–Meier graph for BMI categories and breast cancer in **a** premenopausal women and **b** postmenopausal women the IBIS-I trial

**Fig. 3:**
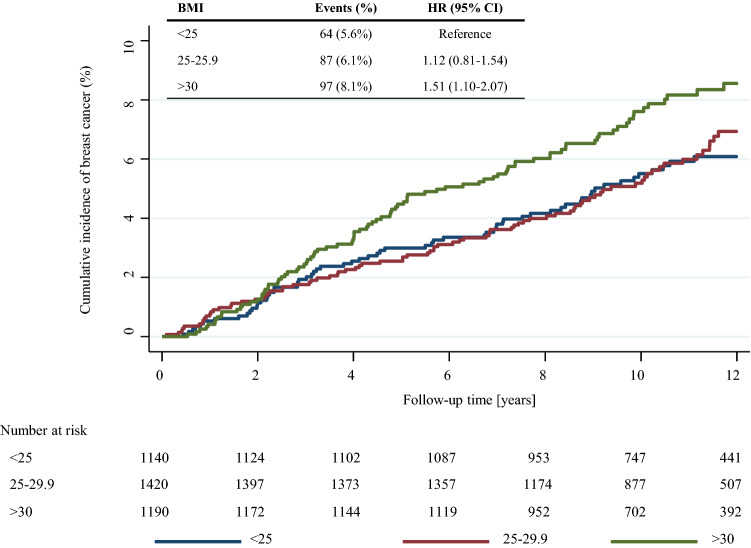
Kaplan–Meier graph for BMI categories and breast cancer postmenopausal women in the IBIS-II trial

When BMI categories were used, breast cancer risk was increased among postmenopausal women with overweight and obesity compared with healthy weight women in both IBIS-I and IBIS-II cohorts (Table 2, Figs. 2b, 3). However, a statistically significant effect was only observed in the IBIS-II trial among women with obesity at baseline compared to those with healthy weight [adjusted HR = 1.51 (1.10–2.08), *p* = 0.01] (Fig. 3). These effect estimates were marginally strengthened after exclusion of ER-negative tumours, but a heterogeneity test was not significant (data not shown).

**Table 2:** Relationship between baseline BMI and incidence of breast cancer among IBIS-I and IBIS-II postmenopausal participants

	BMI (kg/m^2^)	IBIS-I (*N* = 3731)			IBIS-II (*N* = 3750)		
		*N*	Events (%)	HR (95% CI)	*N*	Events (%)	HR (95% CI)
Unadjusted	Continuous			1.15 (1.04–1.27)			1.21 (1.09–1.34)^a^
	< 25.0	1463	113 (7.7%)	Ref	1140	64 (5.6)	Ref
	25.0–29.9	1371	131 (9.6%)	1.26 (0.98–1.62)	1420	87 (6.1)	1.12 (0.81–1.54)
	≥ 30	896	90 (10.0%)	1.34 (1.01–1.76)	1190	97 (8.1)	1.51 (1.10–2.07)
Adjusted^b^	Continuous			1.14 (1.03–1.26)			1.20 (1.09–1.35)
	< 25.0			Ref			Ref
	25.0–29.9			1.23 (0.95–1.58)			1.11 (0.80–1.54)
	≥ 30			1.30 (0.98–1.71)			1.51 (1.10–2.08)

Hazard ratios for continuous estimates are per 5 unit increase in baseline BMI

^a^IBIS-II analyses adjusted for IBIS-I participation

^b^Adjusted for age, HRT use, current or previous history of smoking, IBIS-I participation (IBIS-II analyses only) and menopausal status (IBIS-I analyses only)

The extent to which associations between BMI and breast cancer risk differed by menopausal status was investigated in the IBIS-I cohort. There was no association between BMI and breast cancer risk among premenopausal women [adjusted HR = 0.97 per 5 kg/m^2^ (0.86–1.09), *p* = 0.628], but a significant increase in risk was observed with higher BMI among postmenopausal women [adjusted HR = 1.14 per 5 kg/m^2^ (1.03–1.26), *p* = 0.01]. A test for interaction between BMI and menopausal status was significant (*p* = 0.038). Premenopausal women with obesity had a non-significant decreased risk of breast cancer compared with healthy weight women (Fig. 2a). Postmenopausal women affected by overweight and obesity had an increased risk of breast cancer compared with healthy weight women; however, only the effect estimate for obesity reached statistical significance (Fig. 2b, Table 2).

In both IBIS-I and IBIS-II, the interaction between treatment group and BMI (per 5 kg/m^2^) on breast cancer risk was not statistically significant (Table 3). In IBIS-I, the association between BMI and breast cancer risk was similar among women in the tamoxifen and placebo groups. In IBIS-II, the association between BMI and breast cancer risk was marginally stronger in women in the placebo group compared with the anastrozole group.

**Table 3:** Relationship between baseline BMI and incidence of any breast cancer according to treatment received and trial

	IBIS-I	IBIS-II
	HR (95% CI)	HR (95% CI)
Placebo	1.01 (0.99–1.03)	1.23 (1.09–1.38)
Tamoxifen	1.02 (0. 99–1.04)	
Anastrozole		1.14 (0.93–1.39)
P-interaction	0.62	0.55

Analyses adjusted for age, HRT, current or previous history of smoking, IBIS-I participation (IBIS-II analyses only) and menopausal status (IBIS-I analyses only)

Hazard ratios for continuous estimates are per 5 unit increase in baseline BMI

## Discussion

In this analysis of two large randomised preventive therapy trials, among women at increased risk of breast cancer BMI was positively associated with breast cancer risk among postmenopausal women. Effect estimates were consistent with this observation when BMI was categorised. These observations support the scientific consensus that higher BMI increases postmenopausal breast cancer risk [3, 4, 6, 29, 30]. To further examine the role of weight in breast cancer risk among higher risk populations, behavioural weight loss interventions should be examined within randomised trials with breast cancer endpoints. However, sample sizes may be prohibitive [31].

We did not observe a protective effect of BMI on breast cancer risk among premenopausal women, although there was a possible small protective effect among premenopausal women with obesity. Reports of a protective effect of weight have been observed in younger premenopausal women at population level risk [7, 8, 32], and observational cohorts of women with a family history of breast cancer [33]. The effects of BMI on breast cancer risk by menopausal group were different when comparing the IBIS-I and NSABP trials [15]. A number of factors could explain this. No heterogeneity between BMI and menopausal status was reported in the NSABP-P1 trial, and hence, their result might have been a chance finding. Eligibility criteria for the trials were different, and therefore, the populations under study may not be directly comparable. For example, women in IBIS-I were at lower risk than P1 trial participants; in both IBIS trials, participants were less likely to have obesity and previous HRT usage was higher in the NSABP-P1 trial. Menopausal status was only assessed at baseline for the IBIS-I and NSABP-P1 trials, and therefore, different proportions of women may have become postmenopausal during trial follow-up. Despite the larger sample size available for the NSABP analysis, median follow-up was longer in both IBIS-I and IBIS-II cohorts, likely resulting in higher statistical power.

In the adjuvant setting, there is evidence that overweight and obesity may reduce the efficacy of anastrozole [20–23], but not tamoxifen [18, 19]. Our data are among the first to investigate this topic in the primary prevention setting. We did not find convincing evidence to suggest women with higher BMIs experience differential benefit of preventive therapy. Women are likely to experience benefit from tamoxifen and anastrozole preventive therapy regardless of their BMI. These data also indicate that preventive therapy is unlikely to completely mitigate the excess breast cancer risk among postmenopausal women with a higher BMI. A complementary approach of weight management and preventive therapy may optimally reduce breast cancer risk in this population.

Effect sizes for the relationship between BMI and breast cancer risk were slightly but non-significantly larger when ER-negative tumours were removed. The larger effect between BMI and ER-positive breast cancer is supported by NSABP-P1 and P2 data [15], as well as many studies including mainly women with a general population risk [6, 9–11]. While data were available on tumour characteristics including size, grade and nodal status, sample sizes were too small to conduct meaningful sub-group analysis. Future research using larger cohorts is needed to investigate the relationship between BMI and breast tumour subtypes [7, 9, 11, 12].

This study benefited from a large set of well-characterised samples. The length of follow-up and linkage with national registry data provided a unique opportunity to investigate the relationship between BMI and breast cancer risk in a higher risk cohort. There were also limitations. We used BMI as a weight assessment, calculated from baseline measured height and weight, thereby reducing the reporting bias observed in many studies that use self-report. However, no standard protocol was employed for weight measurement. Alternative anthropometric measures such as waist or body fat measures may be more informative for investigating associations with breast cancer risk, particularly for younger women [34–36]. Our observed effects reflect associations with baseline BMI and baseline menopausal status (IBIS-I), and therefore, do not account for postmenopausal transition, adult weight gain or weight in early adulthood, which may be important factors in breast cancer risk [37]. They may also have been affected by detection bias, whereby lumps in women with overweight or obesity are more difficult to palpate than women with healthy weight. This could result in a delayed diagnosis, therefore, increasing postmenopausal breast cancer incidence. However, national screening data indicate that mammography screening is more sensitive in heavier women [38]. Women with a higher BMI are at greater risk of thromboembolic events [39] and, therefore, may have had to cease tamoxifen treatment more frequently. These women would therefore be expected to have a higher risk of breast cancer as they have experienced less benefit from preventive therapy. However, the lack of relationship between weight and preventive therapy adherence suggests that this is unlikely to be a major factor [40, 41]. Our analysis was on an intention-to-treat basis, and therefore, the small differences in medication adherence between the treatment and control groups may have affected our effect estimates [42, 43]. Weight gain has been observed among women using preventive therapy; however, a previous report using the IBIS cohorts indicated that this generally occurred in the first 12 months of participation and was similar among treatment and placebo arms [44].

In summary, higher baseline BMI was associated with an increased risk of breast cancer in postmenopausal women in two separate trials of women at increased risk of the disease. Among the premenopausal women in IBIS-I, there was no relationship between BMI and breast cancer risk. Women are likely to experience benefit from tamoxifen and anastrozole preventive therapy regardless of their BMI. Women at increased risk of breast cancer should be encouraged to achieve and maintain a healthy weight irrespective of their decision to initiate or decline preventive therapy. Supportive weight control programmes alongside chemoprevention may be required to achieve maximal risk reduction.

## Supplementary Information

Below is the link to the electronic supplementary material.

Supplementary file 1 (PDF 383 KB)

Supplementary file 2 (PDF 162 KB)

## Data Availability

The datasets analysed during the current study are not publicly available as IBIS trial participants are still under the follow-up period. Please contact the corresponding author to regarding reasonable requests for data.

## Code availability

Statistical code is available upon reasonable request.
