# Supplementary material for: The impact of body mass index on breast cancer incidence among women at increased risk: an observational study from the International Breast Intervention Studies
Source: Breast Cancer Res Treat. 2021 Mar 3;188(1):215–23. doi: 10.1007/s10549-021-06141-7 (PMC8233270; doi:10.1007/s10549-021-06141-7)
Supplement: Supplementary file 1 — (PDF 383 KB) [file 10549_2021_6141_MOESM1_ESM.pdf]

Barts and The London
School of Medicine and Dentistry

CANCER RESEARCH UK

IBIS-II (Prevention)

PROTOCOL

An international multi-centre study
of
anastrozole vs placebo
in
postmenopausal women
at
increased risk of breast cancer

Approved

Date _13/11/2019

Professor Tony Howell

Chief Investigator

Date _13/11/2019

Dr Ivana Sestak

Trial Statistician

Independent Trial Steering Committee:

Dr Richard Sainsbury (Chair, Independent member)
Dr Judy Garber (Independent member)
Prof Peter Sasieni (Independent member)
Prof Tony Howell (Chief Investigator, Non-independent member)
Prof Jack Cuzick (Observer)
Dr Nick Zdenkowski (Observer)
Dr Ivana Sestak (Trial Statistician, Observer)

Trial Office:

IBIS-II
Centre for Cancer Prevention
Wolfson Institute of Preventive Medicine
Charterhouse Square
London
EC1M 6BQ
UK

T + 44 (0) 20 7882 3838
F + 44 (0) 20 7882 3886
E ibis@.qmul.ac.uk

Independent Data Monitoring Committee:

As of November 2015, the previous duties undertaken by members of the IDMC are undertaken by the independent members of the Trial Steering Committee.

Contents

Introduction 5
1. Aims 5
1.1 Primary 5
1.2 Secondary 5
1.3 Exploratory 5
2. Background 6
3. Study Design 9
3.1 Outline 9
3.2 Dose and Duration 10
3.3 Participating Centres and Recruitment 10
3.3.1 UK 10
3.3.2 Non-UK 11
3.4 Eligibility 11
3.4.1 Inclusion Criteria 11
3.4.2 Entry Criteria 12
3.4.3 Exclusion Criteria 14
4. Study Procedures 16
4.1 Investigations 16
4.1.1 Mammography 16
4.1.2 Bone Mineral Density (BMD) 17
4.1.3 Spinal Radiograph 17
4.2 Personal and medical details 18
4.3 Samples and Specimens 18
4.3.1 Blood samples 18
4.3.2 Pathology specimens 19
4.3.3 Saliva samples 19
4.4 Sub Studies 20
4.5 Management of Trial Participants 20
4.5.1 Follow-up 20
4.5.1.1 Active Treatment Follow-up (Baseline-Month 60) 20
4.5.1.2 Post-treatment Follow-up Questionnaires (PTQ) - Years 6 – 10 21
4.5.1.4 Post treatment follow up - Digital Registry Data tracking 24
4.5.2 Compliance 30

4.5.3 Treatment cessation ... 30
4.5.4 All Adverse Events ... 31
4.5.4.1 Adverse Events (AE) ... 31
4.5.4.2 Serious Adverse Events (SAE) ... 32
4.5.4.3 UK Registry identified SAEs: ... 35
4.5.4.4 Suspected Unexpected Serious Adverse Reaction (SUSAR) and Serious, Suspected Adverse Reactions (SSARs) ... 37
5. Toxicity and Hazards ... 38
6. Analysis of Data and Statistical Considerations ... 41
6.1 Randomisation ... 41
6.2 Endpoints ... 41
6.3 Numbers of Volunteers and Power ... 42
6.4 Data Monitoring ... 43
6.5 Unblinding the Randomisation ... 43
6.6. End of Trial ... 44
7. Consent and Ethics Committee Approval ... 45
8. References ... 47
9. Appendix ... 51
Appendix 1 – Breast Cancer Prevention Trials ... 51
Appendix 2. Summary of Drug Handling Guidelines ... 53
Appendix 3a. SAE reporting flow chart – open sites ... 54
Appendix 3b. SAE reporting flow chart – registry data ... 55

Introduction

IBIS-II (Prevention) is designed to continue the work started in IBIS-I in determining whether a chemopreventive strategy towards breast cancer is beneficial. IBIS-I was set up to investigate the use of tamoxifen as a preventive agent for women with moderate to increased risk of getting breast cancer. IBIS-II (Prevention) is a randomised double blind control trial but this time will compare anastrozole vs placebo. A companion protocol will compare anastrozole to tamoxifen for women with locally excised ER or PgR +ve DCIS.

This study will be run in accordance with the ICH GCP Guidelines, the principles of which have their origins in the Declaration of Helsinki 2000.

Cancer Research UK is supporting this research study and Queen Mary University of London is the sponsor.

1. Aims

1.1 Primary

1) To determine if anastrozole is effective in preventing breast cancer in postmenopausal women at increased risk of the disease.

1.2 Secondary

1) To examine the role of anastrozole in preventing oestrogen receptor positive breast cancer.

2) To examine the effect of anastrozole on other cancers, cardiovascular disease, fracture rates, and non-breast cancer deaths.

3) To examine tolerability and acceptability of side effects experienced by women on the study. (Met at LPLT – March 2017).

1.3 Exploratory

1) To examine the effect of anastrozole on breast cancer mortality.

Quality of life, cognitive function, bone density and other disease markers will also be studied, but are described in separate protocols.

2. Background

Each year in the UK, there are about 40,000 new cases of breast cancer and approximately 13,000 women die from the disease. Worldwide it is the commonest form of cancer in women with an estimated 700,000 cases and 300,000 deaths annually. Breast cancer is responsible for about 20% of the deaths from cancer in women in developed countries. Many women undergo extensive surgery with subsequent chemotherapy, hormone therapy or radiotherapy, but to date there have been only modest improvements in survival. Early detection and wider use of chemotherapy and hormone therapy in the past two decades have led to a reduction in breast cancer mortality of 25-30% (Peto *et al*., 2000). A further improvement might be gained from yet more effective use of adjuvant therapies. Full implementation of the national screening programme could also lead to similar reductions in mortality. However, neither of these approaches will affect cancer incidence, and mortality rates remain high. It is clear that with currently available treatments, the prospects for making a major impact on the morbidity and mortality from breast cancer lie more in the area of prevention. The national screening programme does however offer the opportunity to identify women at increased risk of breast cancer and is very complementary to the aims of a prevention trial. The screening programme could provide for the routine identification of women at increased risk especially those aged over 50 years.

The success of tamoxifen in preventing recurrence and reducing mortality in established breast cancer, (EBCTCG, 1992, 1998), its ability to prevent or delay a substantial number of new contralateral tumours (Cuzick & Baum, 1985; Fornander *et al.*, 1989; Fisher *et al.*, 1989, EBCTCG, 1998) and its very low toxicity makes it an attractive agent to consider in a preventive setting. Four prevention trials have been conducted to examine its role in prevention (Cuzick et al 2002). All of these trials are compatible with a 30-40% reduction in incidence of breast cancer and a test for heterogeneity is not conventionally significant. Thus tamoxifen's effect in preventing or delaying new breast cancers, at least in the short-term for most moderately high-risk women, is now established. However, the side effect profile including a small excess of endometrial cancer, gynaecological problems and thromboembolic disease is such that tamoxifen should not be considered routine

prophylaxis for women at high risk. Further follow up of the existing chemoprevention trials is needed to determine which women will truly benefit most from tamoxifen prophylaxis.

In North America, future prevention activities are focused on new SERMs (Selective Oestrogen Receptor Modifiers). In the MORE trial (Cummings *et al*., 1999) the new SERM raloxifene produced a larger reduction of breast cancer than was seen for tamoxifen in the North American P1 trial, but breast cancer was not a primary endpoint in this trial, which was primarily designed to examine raloxifene's effect on osteoporosis (see Appendix 1). The effect was only seen for oestrogen receptor positive tumours. A prevention trial in 22,000 postmenopausal women at increased risk, comparing tamoxifen to raloxifene (STAR), which started in 1999, is currently ongoing in the United States.

Aromatase inhibitors are a class of endocrine therapies which act systemically to inhibit oestrogen synthesis in tissues. These compounds prevent oestrogen biosynthesis by inhibiting the enzyme aromatase, which catalyses the conversion of adrenal androgens (androstenedione and testosterone) to oestrogens (oestrone and oestradiol). There has therefore been interest in developing these compounds as potential therapies for hormone responsive breast cancer in postmenopausal women.

In postmenopausal women, oestradiol is produced primarily from the conversion of androstenedione to oestrone through the aromatase enzyme complex in peripheral tissues. Oestrone is subsequently converted to oestradiol. Reducing circulating oestradiol levels, either by the use of gonadotrophin releasing hormone (GnRH) agonists in premenopausal women (Rutqvist 1999; Jakesz, *et al.*, 1999) or by the use of aromatase inhibitors in postmenopausal women (Buzdar *et al*., 2000), has been shown to produce a beneficial effect in women with breast cancer.

Aminoglutethimide was the first aromatase inhibitor to be approved for the treatment of breast cancer and has proven efficacy in postmenopausal women with advanced breast cancer (Stuart-Harris *et al.*, 1984). Wider use of the drug has been limited by its lack of specificity, resulting in a requirement for concomitant administration of corticosteroids, and the occurrence of troublesome side effects (Wells *et al.*, 1978). Consequently, research has been focused on the development of aromatase inhibitors with greater specificity and a better tolerability profile.

The new third generation aromatase inhibitors have shown good efficacy in advanced breast cancer and have a very low toxicity profile. They offer another approach to local control, prevention of recurrence and the prevention of primary breast cancers, which may be superior and/or complimentary to the use of SERMs.

Anastrozole is a potent new non-steroidal aromatase inhibitor which is highly selective, well tolerated and effective in treating advanced breast cancer (Buzdar *et al.*, 1997; Jonat *et al.*, 1996; Plourde *et al.*, 1995; Nabholtz *et al*., 2000). In postmenopausal women, a daily dose of 1 mg anastrozole produces oestradiol suppression of greater than 80% using a highly sensitive assay (Geisler *et al*., 1996). Anastrozole does not possess progestogenic, androgenic or oestrogenic activity.

In controlled clinical trials in advanced tamoxifen resistant breast cancer, anastrozole at the daily dose of 1 mg has demonstrated superior clinical efficacy to a standard second line hormonal treatment with megestrol acetate (Buzdar *et al*., 1998). Recent trials of anastrozole as first line treatment in advanced breast cancer suggest that it is as good as and possibly superior to tamoxifen. In particular, Nabholtz *et al*. (2000) reported that anastrozole may be more effective than tamoxifen as first line treatment for ER-positive advanced breast cancer (hazard ratio 0.69, *P* = 0.005 for time to progression in one study with mostly ER-positive tumours only; no difference overall in a second study (HR = 1.0), but HR = 0.87 in ER-positive patients in that study). A recent update of the combined trials (Buzdar, *et al.* 2000) showed that time to progression was significantly increased with anastrozole in patients with receptor positive tumours (median 10.7 vs 6.4 months, *P* = 0.02). Toxicity has generally been similar or lower (especially for thromboembolic disease and vaginal bleeding) in the anastrozole arm.

A very large trial (ATAC) is currently evaluating the role of anastrozole both alone and in combination with tamoxifen compared to tamoxifen in the adjuvant setting for early breast cancer. This trial has recruited 9366 patients and more than 1000 recurrences or deaths have now been recorded. After a median follow-up of 33 months, the initial results have been reported (ATAC Trialists, 2002). Significantly fewer recurrences have been reported on the anastrozole arm compared to tamoxifen (HR=0.83, p=0.013). New contralateral tumours were also reduced by 58% (P= 0.007) compared to tamoxifen. Given that tamoxifen can reduce new tumours by 30-40%, this suggests that anastrozole may produce a potential reduction of 70-80% in primary breast cancer in the prevention setting. The side effect profile is generally favourable, with fewer endometrial cancers,

thromboembolic events, strokes and hot flushes than tamoxifen (Table 5, Section 4). However, there are significant increases in musculo-skeletal disorders (primarily arthralgia) and fractures in the anastrozole group when compared with the tamoxifen group (Table 5). Thus there is a strong case for evaluating the role of anastrozole in the prevention of breast cancer.

As of April 2002, the total exposure to anastrozole exceeded 550,000 patients-years (personal communication, T. Sahmoud, AstraZeneca) and this is increasing rapidly. A review of the clinical trial safety database did not reveal evidence of clinically significant interactions in patients treated with anastrozole who also received other commonly prescribed drugs.

See Appendix 1 for further details on results of other breast cancer prevention trials.

3. Study Design

3.1 Outline

A multicentre, randomised placebo-controlled clinical trial of 6,000 (however, as of July 2010 and at point of LPLT sample sizes were re-calculated – see section 6.3) postmenopausal women aged between 40 and 70 years who are at increased risk of breast cancer will be conducted.

The specific categories for eligibility are shown in Section 3.4. In general terms increased risk is determined from family history, previous benign disease with evidence of proliferation, mammographic dysplasia, and nulliparity. All women will have a mammogram taken to exclude breast cancer before randomisation and a blood sample will also be taken for analysis of biochemical and other risk factors for breast cancer and potential side effects.

A parallel trial of 4,000 women with oestrogen or progesterone receptor positive ductal carcinoma in-situ (DCIS) who have received adequate local treatment will also be conducted in the same way except that they will be randomised between two active treatment arms (tamoxifen vs. anastrozole, see separate IBIS-II (DCIS) Protocol).

3.2 Dose and Duration

Women will be randomised in a 2-arm design to receive one of the following:

1. Anastrozole 1mg
2. Anastrozole placebo

Randomisation will be stratified by major (hub) centre. Randomised blocks will be used to maintain balance. Randomisation will be performed centrally by electronic contact with the main trial centres in the UK and will be available at all times. All treatment will be on a daily basis for 5 years. Tablets will be supplied in numbered, light-proof containers containing a 6-month supply. Each container will have extra tablets to allow for some delay in the next appointment. See Appendix 2 for further details of drug handling procedures.

Physicians and nurses must attempt to determine that compliance is likely to be good before randomising subjects.

3.3 Participating Centres and Recruitment

All centres will randomise through the central office in London via electronic data transfer. The database is securely located on an Oracle server hosted on the network at the IBIS-II CCO. Back-up systems will be provided for randomising by fax and failing that, by telephone or e-mail. The remote randomisation will be part of the standard trial software distributed to all centres for the data management of the trial.

3.3.1 UK

Recruitment will be based at regional centres. Regional centres will enter patients at their own hospital and also serve as a local co-ordinating centre (hub) for satellite centres in nearby hospitals throughout the region. Each centre will be expected to recruit an agreed number of new participants per year to reach their target.

All UK centres are responsible for collection and management for blood samples and other specimens obtained from their participants as specified in the protocol and site contract.

3.3.2 Non-UK

All non-UK centres are responsible for collection and management of blood samples and other specimens obtained from their participants. Data and specimens will be transferred to the central office at regular intervals as specified in the protocol and site contract.

Where possible other international centres will be managed through existing national or international trial groups.

3.4 Eligibility

3.4.1 Inclusion Criteria

a) All women must be postmenopausal and between the ages of 40-70. In certain circumstances women who are within 24 months of this age range may join the trial by obtaining prior approval from one of the IBIS-II steering group co-chairmen. Postmenopausal status is defined as meeting one or more of the following criteria:

1. over the age of 60
2. bilateral oophorectomy
3. aged ≤ 60 with a uterus and amenorrhoea for at least 12 months
4. aged ≤ 60 without a uterus and with FSH >30 IU/L. NB If participant is taking HRT, an 8 week wash-out period is required prior to FSH test being performed.

Assessment of post-menopausal status is ultimately the responsibility of the PI/clinician involved in the participants' care.

b) Women must satisfy at least one of the entry criteria listed in section 3.4.2 below.

A mammogram must have been taken within the last year and must not show any evidence of breast cancer.

c A baseline bone mineral density scan within the last two years (DXA either of hip, lumbar spine, femoral neck or forearm) will be required for all women. Two spinal x-rays in one lateral dimension will be required to assess low trauma vertebral fractures.

d) Fully informed signed consent must have been obtained.

e) Participants treated for Hodgkin's Disease are eligible if they develop the disease before the age of 30 and have been treated with mantle radiotherapy.

f) Participants who took part in IBIS-I but have been off trial therapy for at least 5 years.

3.4.2 Entry Criteria

Entry criteria will be age-dependent to reflect increasing baseline risk with age.

Women aged 45-70

The entry criteria are based on a relative risk of at least two-fold and are similar to those in IBIS-I.
At least one of the following must be satisfied:

1) First degree relative who developed breast cancer at age 50 or less
2) First degree relative who developed bilateral breast cancer
3) Two or more first or second-degree relatives who developed breast or ovarian cancer. If both relatives are second degree and on opposite sides of the family, then at least one must have been diagnosed at age 50 or less
4) Nulliparous (or first birth at age 30 or above) and a first degree relative who developed breast cancer
5) Benign biopsy with proliferative disease and a first degree relative who developed breast cancer

   Mammographic opacity covering at least 50% of the breast in absence of HRT use within the last 3 months. Films or digitised images **must** be verified by either a designated national radiologist or by someone that has undertaken the mammographic density training for confirmation of eligibility prior to randomisation.

Also women aged 60-70

Because of their higher baseline risk, women aged 60-70 can enter the study with a smaller relative risk (approximately 1.5 or greater). This corresponds to a similar 5-year absolute risk as that for a 50-year old woman in the above group (approximately 3% at 5 years). These women need only have one or more of the following risk factors:

6) First degree relative with breast cancer at any age
7) Age at menopause ≥ 55 years
8) Nulliparous or age at first birth 30 years or above

Women aged 40-44

Women aged 40-44 who are postmenopausal (usually because of a bilateral oophorectomy) are eligible if they satisfy one or more of the following criteria (approximately 4-fold risk or greater):

9) Two or more first or second degree relatives who developed breast or ovarian cancer at age 50 or less
10) First degree relative with bilateral breast cancer who developed the first breast cancer at age 50 or less
11) Nulliparous (or first birth at age 30 or above) and a first degree relative who developed breast cancer at age 40 or less
12) Benign biopsy with proliferative disease and a first degree relative who developed breast cancer at age 40 or less

All Age Groups (40–70) – women who have had certain breast conditions will also be eligible. These are:

14) Lobular carcinoma in situ (LCIS)
15) Atypical ductal or lobular hyperplasia in a benign lesion
16) Ductal carcinoma in-situ (DCIS), treated by mastectomy within the last 6 months, Oestrogen Receptor or Progesterone Receptor status, (ER or PgR), of DCIS must be known, and must be greater than or equal to 5% positive cells. This is equivalent to a Quick score of 3 or above and an H-score of 10 or above. Quick scores and H-scores must be given as whole numbers. DCIS patients

treated by mastectomy with single or multiple focus of microinvasion (<1mm) are eligible for the Prevention trial. An extension of up to a further 3 months may be obtained for this eligibility criterion with prior approval from one of the IBIS II Steering Group co-chairmen.

17) Women with a ten-year risk greater than 5%, who do not fit into the above categories (risk equivalent). All risk equivalent women must be approved by the Steering Committee Co-Chairman (London IBIS central office). These women must have clearly apparent family history and/or other risk factors indicating appropriate increased risk of breast cancer for their age. Particularly careful assessment of the risk-benefit for these women should be undertaken before a woman from this group is entered.

In all cases, if desired, an individual clinician may enter only a subset of the eligible women.

3.4.3 Exclusion Criteria

a) Premenopausal women.

b) Any previous diagnosis of breast cancer (including DCIS excised >6 months ago)

c) Any other previous cancer in the past 5 years (except non-melanoma skin cancer or in situ cancer of the cervix).

d) Current or previous tamoxifen or raloxifene or other SERMs use for more than 6 months or participation in IBIS-I. However, women who took part in IBIS-I and have been off trial therapy for at least 5 years are eligible.

e) Intention to continue to use oestrogen-based hormone replacement therapy (HRT).

f) Women who have either had a prophylactic mastectomy or are planning to have this procedure.

g) Evidence of osteoporosis* or low trauma vertebral fractures within the spine. However, these women may be eligible if their T-score is greater than or equal

to minus four, and if they have no more than two low trauma vertebral fractures. In either case they must be managed in accordance with local clinical procedures for treatment of such women ie take bisphosphonate treatment and have regular DXA scans. Women with T-scores less than minus four, or with more than two low trauma vertebral fractures are not eligible.

* The WHO definition of osteoporosis is a T-score of -2.5 or less at the femoral neck. However, it is recommended that a T-score of -2.5 or less at the lumbar spine should also be considered as osteoporotic in terms of all IBIS-II protocols.

h) Any severe concomitant disease that would, in the opinion of the investigator, place the woman at unusual risk or confound the results of the study. Reference should be made to the appropriate Summary of Product Characteristics (SmPC).

i) Life expectancy of less than 10 years or other medical condition that would significantly interfere with the ability to accept the chemopreventive treatments.

j) Psychologically and physically unsuitable for five years anti-oestrogen therapy.

k) Treatment with non-approved or experimental drug during the 6 months before randomisation.

l) History of gluten and/or lactose intolerance.

Systemic oestrogen replacement therapy is not allowed whilst women are taking trial medication. If serious menopausal symptoms develop during treatment, the following approach should be adopted:

1. Non hormonal complementary therapies may have some beneficial effect and some women may achieve relief from dietary and lifestyle changes.

2. Non hormonal treatment of specific symptoms, e.g. venlafaxine may help with hot flushes. Senselle (a water based, non-hormonal vaginal lubricant) may alleviate vaginal dryness. Following the report of the Million Women Study (Beral, 2003) progestagens and tibolone are contra-indicated for women at increased risk of breast cancer. Please note that this is not an exclusion

criterion for IBIS-II but women should be made aware of this risk and a clinical decision should be made as to inclusion on the trial.

3. In cases of serious vaginal discomfort low dose oestrogen preparations may be used for as short a period as possible.

4. If the above are unsuccessful, trial medication can be reduced to alternate days. If symptoms persist, a treatment holiday can be tried next.

5. If unacceptable symptoms persist and oestrogen-based therapy is deemed necessary, then trial medication must be stopped. If, at a later stage, HRT is stopped, then trial medication can recommence.

Each of the above options must be clearly documented in the participants' records. Breaks from trial medication should be kept to a minimum, ideally no longer than 1 to 3 months.

4. Study Procedures

4.1 Investigations

4.1.1 Mammography

All women will have a physical examination and mammogram (and where necessary fine needle cytology or biopsy) to exclude pre-existing malignancy. A clear mammogram within the twelve months before entry will be accepted. If there is a delay in receiving the test results, an extension of up to 6 months may be obtained with prior approval from one of the IBIS II Steering Group co-chairmen. The date and identification number of all mammograms should be recorded to facilitate access to mammograms for central review purposes. During the follow-up period mammography will be performed at least once every 2 years. The exact frequency will be decided locally, and as considered necessary for clinical reasons. The IBIS-II CCO in London will request these mediolateral (ML) or mediolateral oblique (MLO) mammograms of both breasts from centres or they may be sent as applicable (but should request authorisation from the IBIS-II CCO before doing so). If film based mammography is performed, the original films will be required. If the

mammography is performed using direct digital machines, then the digital file may be submitted. In cases where a mammogram is not available or a magnetic resonance image has been performed, the MRI will be accepted instead. The IBIS-II CCO will co-ordinate the collection, digitisation and return of requested mammograms using tracked courier services from sending centres. Mammograms will not be required to be collected during the post treatment follow-up phase (beyond 5 years after randomisation).

4.1.2 Bone Mineral Density (BMD)

A baseline DXA scan within the last two years will be required for all women prior to randomisation. If there is a delay in receiving the test results, an extension of up to 6 months may be obtained with prior approval from one of the IBIS II Steering Group co-chairmen. The DXA scan can be performed at any site and use of portable DXA machines that examine the forearm are acceptable. A detailed sub study will investigate bone parameters in greater detail in selected centres. In these centres bone mineral density measurements will be taken in approximately 1,000 women by DXA scans at baseline, and years 1, 3, 5, and 7 to examine the changes in BMD during and after treatment. Full details of investigators and procedures can be found in the separate bone sub study protocol. (Please note: as of Sept 2010 the bone sub-study is closed for recruitment.)

4.1.3 Spinal Radiograph

A baseline radiological assessment must be carried out within two years prior to randomisation to rule out low trauma vertebral fractures. If there is a delay in receiving the test results an extension of up to 12 months may be obtained with prior approval from one of the IBIS II Steering Group co-chairmen. The spinal radiograph requires two x-rays in one lateral dimension to view the thoracic and the lumbar vertebrae. These must be reported on by a qualified radiologist and the report must be available before randomisation takes place. If there are more than **TWO** low trauma vertebral fractures, then the woman cannot take part in any of the IBIS-II protocols. (The European Prospective Osteoporosis Study (Epos) Group, 2002).

Note: If the spinal x-ray report states that there is wedging of more than 20% then this should be considered equivalent to one fragility fracture.

4.2 **Personal and medical details**

A questionnaire giving height, weight, smoking habits, selected clinically relevant medical history and risk factors for breast cancer must be completed for all women entering the study – entry Case Report Form (CRF).

Information on prescriptions and hospital visits (other than those directly associated with the study) will be recorded in order to facilitate an assessment of additional costs or savings to the health service related to long term use of tamoxifen or anastrozole. It is difficult to estimate what the outcome of this analysis will be at this stage, since the costs of diagnosing and treating some side effects may be more than offset by reductions in other areas.

4.3 **Samples and Specimens**

4.3.1 Blood samples

Women will have a sample of 14ml (2 x 5ml SST tubes and 1 x 4ml EDTA tube) venous blood taken at entry and 10 ml (2 x 5ml SST tubes only) will be taken at years 1 and 5 as clotted whole blood. 4ml of the entry sample will be put into the EDTA tube provided and used to look for susceptibility genes to breast cancer or side effects. In the UK, both tubes will be posted immediately by first class postage in pre-addressed SARSTEDT t-box containers to St. Mary's Hospital, London. All samples except the EDTA sample must be spun down locally before posting. Follow-up samples will be treated identically except that the EDTA tube will not be required. Bloods will be stored at -70ºC and analysed retrospectively, mostly on a case-control basis in an anonymous fashion. They will be used to evaluate biomarkers, which are potential but not yet established markers for risk of disease or response to the preventive therapies under study. The follow-up blood samples will also be used to monitor compliance in a sample of women. The blood samples will remain the property of the Steering Group.

Arrangements for non-UK centres will be made in collaboration with the IBIS Co-ordinating Centre on an individual basis, with frozen aliquots being sent in dry ice to London for long-term storage on a regular basis as agreed at the site initiation visit and in the site contract.

4.3.2 Pathology specimens

A set of representative diagnostic H&E stained slides plus the original hormone receptor assay slides (ER and/or PgR) will be required for central review from those patients entering the study after mastectomy for DCIS (entry criterion 16). Paraffin blocks containing representative areas of the tumour will also be requested. The latter will be used to make additional slides and tissue arrays, and will be returned to the sender. If possible, the pathologist should be made aware of the woman's involvement in the IBIS-II Study before blocks are taken. It may then be possible to make extra blocks and slides specifically for study purposes. If blocks are not available, 10 unstained sections on uncoated slides should be provided. A standard DCIS Pathology Entry Form must be completed for each specimen and this is included in the case report forms. In addition, a copy of the original pathology reports (and a copy of the UK Sloane form, where available) must be provided. Diagnostic slides and paraffin blocks will be requested for all breast, endometrial, or ovarian cancers developing after trial entry. These samples will be used for central pathology review and marker studies, and will remain the property of the Steering Group, who will be responsible for deciding how they will be used in any further projects.

4.3.3 Saliva samples

Women who did not provide a baseline EDTA blood sample should be approached to request a saliva sample used to look for susceptibility genes to breast cancer or side effects. There is a separate Patient Information Sheet and Consent Form for this. The IBIS-II CCO will inform sites of eligible participants to this part of the protocol and sites will be asked to call participants to inform them of this request and obtain verbal consent. If the participant agrees to this, sites will send out a saliva kit to be collected at home. Together with the kit, the PIS and CF for this process will be sent to the participant along with instructions for providing the saliva sample. Participants will be asked to read and keep the PIS, sign and date the CF and post it with the saliva sample to the IBIS-II CCO in the freepost addressed pack provided.

The saliva samples will be analysed for the same biomarkers as the EDTA blood samples and will remain the property of the Steering Group.

4.4 Sub Studies

Measurements of changes in hormone levels, lipid profiles, bone biomarkers, and endometrial biopsies will be made in selected centres and will be considered as separate studies and are not addressed in this protocol. Data collection will be kept at a minimum and only items that are essential to analyse the specific endpoints listed below will be collected. All protocols for special studies will be submitted to the Steering Committee for information and co-ordination. A special detailed study on the quality of life and cognitive function will also be undertaken in selected centres. These studies will require appropriate Ethics Committee approval.

4.5 Management of Trial Participants

4.5.1 Follow-up

4.5.1.1 Active Treatment Follow-up (Baseline-Month 60)

Women will be seen in clinic at six months and one year in their first year of participation. Then they may be seen annually with an option to attend for a six monthly visit as well. All women may also be contacted by their Local Coordinator every six months to offer ongoing support and information as necessary during the five years of treatment, but no data is required for the study at these contacts.

Advice on maintaining healthy bones (e.g. calcium and vitamin D supplementation, sunlight and weight bearing exercise) will be offered to all women in the trial. Treatment with bisphosphonates should be considered where necessary. The use of raloxifene or any other SERM, or HRT for osteoporosis or any other reason is not permitted in this trial. Interested general practitioners (GPs), and those within the vicinity of a centre, will be sent the study protocol and information leaflets, as these GPs will be very important both for recruitment and for support during the study, which should help with compliance. Blood samples (10ml) to document changes in biomarkers and to monitor compliance will be taken at year 1 and year 5. Mammograms will be taken at intervals, as described in section 4.1.1. These will be reviewed centrally on a subset to examine the effects of treatment on mammographic opacities.

4.5.1.2 Post-treatment Follow-up Questionnaires (PTQ) - Years 6 – 10

After 5 years of active treatment, women will continue to be followed up for clinical events of interest including SAEs, via the 'post-treatment follow-up' CRF for a further 5 years. These will specifically ask the patients about the following events: -

- Breast cancer and DCIS (endpoint not requiring reporting)
- Stroke or TIA
- Other cancers (except basal cell carcinoma, squamous cell carcinoma of the skin and carcinoma in situ of the cervix)
- Cardiovascular events e.g. angina, heart attack, hypertension
- Thromboembolic events e.g. deep vein thrombosis, pulmonary embolism, phlebitis
- Osteoporosis/Broken/fractured bone
- Deaths

The decision to collect events of interest rather than asking about all AEs is based upon the following

- 5x half-life of IMPs will be reached within 35 days i.e. a short time period and therefore long term monitoring is not required.
- Except for certain conditions (endometrial cancer, osteoporosis/fracture, cardiovascular disease), further side-effect either stop with IMP washout or are of negligible significance.
- The safety profiles of Anastrozole has already been well-established across multiple trials and long follow up periods (as of 31 July 2016, the total cumulative exposure figure for ARIMIDEX in the marketed setting is calculated to be in excess of 8 million patient years)
- This is an ageing trial population with many AEs meeting the definition of SAEs but that are not of direct relevance to the trial objectives. To date, approximately 6% of reported SAEs in the post-treatment phase have been defined as causally related by the site investigator. Efforts should be re-focused on the collection of events known to be IMP related and the identification of any emerging safety signals. A more defined scope of AE collection is therefore warranted.

- All identified SARs/SUSARS will meet expedited reporting timeframes.

Sites have the option to conduct this by clinic, phone, postal questionnaire or if the patient is not able to be contacted, consultation of hospital records. This will collect data required to meet the primary, secondary and exploratory objectives of the trial.

Prior to the implementation of amendment 60, the above procedures were completed at all sites. Following the implementation of amendment 60, open international sites continue to follow this procedure, while patients from closed UK and international sites are followed up via digital registries as described in section 4.5.1.4.

4.5.1.3 Post- treatment Follow-up Questionnaires (PTQ) - beyond 10 years

The 16 year follow-up results from the IBIS-I Trial (Cuzick et al, 2015) was seminal in determining the long-term benefits of tamoxifen as prophylaxis for breast cancer. This confirmed that "the risk-reducing effect of tamoxifen appears to persist for at least 10 years, but most side effects of tamoxifen do not continue after the 5-year treatment period". Collection of breast cancer occurrence for extended follow up periods (beyond 5 years) will be pivotal in determining if the effect of anastrozole, like tamoxifen, can persist for at least 10 years.

The ATAC trial 10 year follow-up results (Cuzick et al., 2010) extended the previously reported superior efficacy of anastrozole over tamoxifen. Furthermore, results demonstrated a carryover benefit for recurrence in the hormone-receptor positive population, which is larger than that previously shown for tamoxifen. This highlights the need for continued long term follow-up in IBIS-II, collecting breast cancer occurrence data up until the end of trial definition in 2022. Published results from this trial (Cuzick *et al.* 2014) have already been published at median average follow up of 5 years (IQR 3·0–7·1), 40 women in the anastrozole group (2%) and 85 in the placebo group (4%) had developed breast cancer (hazard ratio 0·47, 95% CI 0·32–0·68, p<0·0001). Sustained

follow up is required to see if this difference persists and in the case of similar tamoxifen trials, may further diverge.

Sites should continue to follow-up those participants that have reached 10 years of follow-up beyond this time in the same way as for years 6 – 10 (section 4.5.1.2). Data will be collected on events of interest to meet primary and secondary endpoints as described in section 6.2.

Participants have been contacted by their trial site to seek their consent for follow-up beyond 10 years. This should be collected at the last PTQ visit at the year 10 post-treatment visit. Consent will be documented via completion of an electronic CRF at the trial site and can be done verbally on the phone or by paper CRF if the site posts the questionnaires for completion. The post-10-year phase of the IBIS-II trial should be considered as an additional observational element to the trial. Key safety data will nevertheless be collected as described before and reviewed as part of final study analysis, and forwarded to AstraZeneca.

Prior to the implementation of amendment 60, the above procedures were completed at all sites. Following the implementation of amendment 60, open international sites continue to follow this procedure, while patients from closed UK and international sites are followed up via digital registries as described in section 4.5.1.4.

4.5.1.4 Post treatment follow up - Digital Registry Data tracking

In the UK, digital registries (NHS Digital, Public Health England (PHE), Northern Ireland Cancer Registry, National Welsh Informatics Service, NHS Services Scotland) will be used to collect follow up information relating to the cohort from month 60 until the end of their participation in the trial. These registries will provide data on cancer incidence and mortality which utilises in-patient and A&E data and national cancer registry data.

Data collection and storage

Data will be collected annually via the UK-based digital registries and as negotiated with the international sites (where applicable). No data will be exported outside the EU or the UK. Data collected from international sites will be stored according to the terms outlined in the DSA.

Long-term follow-up data received from national registries will be processed and held on a secure Barts Cancer Centre IT network. Data registry variables specified in Table 1 will be used to capture breast cancers, deaths, other cancers, cardiovascular, cerebrovascular, and fractures to meet the study objectives. Supplemental information on tumour markers will be collated from national and devolved cancer registry datasets (Table 2).

Table 1. Events of Interest/ICD code

**Event**	**ICD-code**
Breast cancer	
Breast cancer (invasive)	C50.0-9
Breast cancer (DCIS)	D05.0/1/7/9
Other malignant cancer	C00-43, C45-49 and C51-97 (all sub-codes)<br>This includes all other cancers except skin
Other events	
Pulmonary embolism	I26.0, I26.9
Deep vein thrombosis	I80.2
Cerebrovascular events	I63 (all sub-codes), I64
Cardiac arrest	I46 (all sub-codes)
Acute myocardial infarction	I21 (all sub-codes), I22 (all sub-codes)<br>I22 is for subsequent myocardial infarctions
Heart failure	I50 (all sub-codes)

Transient cerebral ischaemic attack	G45.8, G45.9
Hypertension	I20, I15 (all sub-codes), I27 (all sub-codes)
Fractures	M80 (all sub-codes), T02.9, T08, T10, T12, S02 (all sub-codes), S12 (all sub-codes), S22 (all sub-codes), S32 (all sub-codes), S42 (all sub-codes), S52 (all sub-codes), S62 (all sub-codes), S72 (all sub-codes), S82 (all sub-codes), S92 (all sub-codes)

Table 2. Cancer data variables from Cancer Registries (PHE and NICR)

**Data item**	**Relation to study objective**
NHS number	Required for record-level data to be linked to specific individuals in our cohort
Date of Birth	Required for record-level data to be linked to specific individuals in our cohort. Survival analysis. To determine the effectiveness of the intervention.
Date of death of the patient	To determine if intervention was successful in preventing cancer incidence
Cause of death	
Diagnosis date	
Diagnosis death certificate only	Primary endpoint confirm participants cause of death
Site of neoplasm	Study endpoint to identify location of cancers
Site of the cancer	
Histology code	
Grade of tumour	Study endpoint to confirm full details of all cancers
Size of the largest dimension of the tumour, in mm	
Number of nodes excised	
Number of nodes involved	
Laterality	
Oestrogen receptor status of the tumour	
Oestrogen receptor score of the tumour.	
Progesterone receptor status of the tumour	
Progesterone receptor score of the tumour	
HER2 status of the tumour	
Excision margin	
Screen detected cancer	

Code for the Trust at diagnosis	Trace origin of diagnosis potentially query Hospital for furthe details
Name of the trust at diagnosis	

<u>UK Registry data sources</u>

Datasets received from the national registries are a compilation of information collected by the following national data service entities: HES (provides the following datasets for Admitted Patient Care and Accident & Emergency Care), National Cancer Registry, Civil Registration Mortality data, and PHE. Tables 3 and 4 show what data will be received from the different national registries.

Table 3. Digital registry sources of variables

Data source	Variables	Information provided
Hospital Episode Statistics (HES) data set variables	GP Code<br>Date of diagnoses<br>Date of diagnoses<br>Description	GP contact details<br>Cardiovascular and cerebrovascular events, new and recurrent breast cancers other cancers, bone fracture…
Cancer registry data set variables	Date of diagnoses<br>Diagnoses Code<br>Description	New and recurrent breast and other cancers including melanomas
Civil Registration Mortality data set variables	IBIS-II<br>Status<br><br>Date of<br>Death<br><br>Cause of death	Death and cause of death
Public Health England data set variables	Cancer registry - patient table<br>Cancer registry - tumour table	Supplemental data on tumour markers. TNM staging and grading, ER, PR and HER status of breast cancers

Table 4. Current digital dataset provision Oct 2019*

Organisation	Devolved UK Nation	HES	Mortality	Cancer Registry	Tumour markers
NHS Digital	England/Wales	x	x	x	
NWIS (NHS Wales Information Service)	Wales	x			
NICR (Northern Ireland Cancer Registry)	Northern Ireland	x		x	x
NSS (NHS National Services Scotland)	Scotland	x		x	x
PHE	England			x	x

*The organisation providing data could change throughout the course of the study and a copy of this table reflecting current practice will be maintained in the IBIS-II data management plan.

<u>Receiving Data from Digital registries</u>

The original raw data sets are received from the data registry provider through Data Access Request Service (DARS) online for NHS Digital and other digital registry datasets. Once received, the files are saved in a restricted access folder on the Barts Cancer Centre network and imported into Oracle according to the IBIS-II data management plan (DMP).

<u>Unmatched patient details</u>

If an IBIS-II participant's details are incorrect, out of date, or not on file, it may not be possible to ensure a correct match with the relevant digital registry records. If matching fails, effort will be made to identify why and unmatched events should be resolved where possible. If the site is open when the event is identified the team should contact the participant's previous local site if feasible or if the sites is closed, their GP, if details are known.

<u>Patient identifiable data and raw extracts</u>

PID including the participants full name, NHS number, date of birth and postcode is collected by the sites and stored on an encrypted table within the main study database. Section 7 details the consent procedure in place for collection of PID.

Raw data extracts received from the digital registries will either be sent linked to the IBIS-II study number and PID removed by the data registry beforehand or it will be sent containing PID. This will depend on the specific policy of the data organisation.

When PID is sent to the IBIS-II research team from a data registry, a suitably qualified person will pseudonymise the datasets by linking the data to the existing IBIS-II dataset and remove all PID prior to further processing by the IBIS-II data management team to minimise unmerited access to PID All raw datasets are considered source data and should be stored for the duration of the study and archiving period. This data will be stored on an encrypted, un-networked computer at the Wolfson Institute of Preventive Medicine,
The ICD-10 code list of events (Table 1) will be used to match events with further details on deaths, new or recurrent breast cancers from variables listed in Table 2.

Following up with GPs
Follow up with participants' GPs for more information about new or recurrent breast cancers reported via the digital registries will occur if the information provided is not sufficient or where tumour marker data is not available (e.g. Wales).

Additional information obtained from GPs responses should be logged and linked to the SNO only and entered in the IBIS-II database. The source of the data should be specified on the Oracle tables.

Participant withdrawal
Participants can withdraw from flagging at any time. Participants can also request communication from email only. However, data will not be removed from any analysis that has already been completed and participants have previously been informed of this.

Participants can request to withdraw from further data collection by contacting the IBIS-II team either by phone or completing the online form via the IBIS-II website (https://www.ibis-trials.org/healthsurveillance). The IBIS-II email account is monitored on a regular basis by

the IBIS-II team and actioned accordingly. The participant status change will be recorded on the main oracle table according to the IBIS-II DMP. Participants will be notified by email or letter within 10 working days that their request to withdraw has been processed and they are no longer being followed up.

Patients lost to follow up

If no data are received from NHS Digital because either ‘no trace’ or type 2 objections are upheld, the participant will not be immediately considered lost to follow up for this study. Instead, if data is not collected for a participant in any given year, they will remain on the list of participants for which data is requested until either primary outcome data have been collected or the IBIS-II team considers that sufficient information has been collected to report the final results of the study. If no data further has been reported, they will be reported as lost to follow up in the final analysis.

Scope of UK registry data collection

Digital registry flagging is used for the UK cohort for the full duration of follow up provided valid consent (or CAG exception) is in place as described in section 4.5.5.3. While a UK site is open, registry data will be collected to confirm and underpin site-reported events. Once the site is closed, long term data collection will transition to data collection via the UK digital registries only.

HES covers all NHS Clinical commissioning groups (CCGs) in England and Wales including private patients treated in NHS hospitals, patients’ resident outside of England under care provided by treatment centres, including the independent sector, funded by the NHS. For participants who commenced the study in England and moved to either Northern Ireland or Scotland, cross-border transfers will be recorded so they can be followed up via the appropriate national registry (NSS or NICR). Similarly, if participant moved from the devolved nation to England this will also be detected.

4.5.2 Compliance

If women are concerned about a symptom potentially related to trial medication, it is preferable to consider trying a dose reduction, i.e. alternate days, or offer a 'treatment holiday' rather than to stop treatment altogether. Full treatment can be restarted after an appropriate interval (usually 1-3 months) or a decision about withdrawal can be made subsequently if symptoms persist. Treatment holidays can also be used in other special circumstances, but should always be documented on the follow-up form, as should changes in daily dose.

4.5.3 Treatment cessation

Adherence to treatment protocol will cease if any of the following circumstances arise:

i) the woman develops breast cancer (including DCIS)
ii) her T-score drops below minus four
iii) she is found to have a new low trauma vertebral fracture.
iv) development of any new condition/disease where it's necessary treatment contraindicates trial therapy or associated treatments.

The trial medication will be stopped and the woman should be referred for treatment according to local practice. Should a woman develop any concurrent condition, for example heart disease, during the study, it would not be considered necessary for her to leave the study. However, as with any illnesses that develop during the woman's participation in the study, the decision about continuing will rest with the clinician involved, the woman herself and her general practitioner.

Following the positive results of the first analysis (Cuzick et al, 2014), if a participant decides to request a codebreak and is on anastrozole, she will be able to continue the remainder of the trial treatment unblinded. An SOP will be available for pharmacists for procedures on preparing subsequent IMP supplies to be open-label. Participants will continue with follow-

up visits as when blinded to treatment. Should a participant decide to request a codebreak and is on placebo, she will no longer receive trial medication but they will be advised to discuss further with their local investigator / GP to identify possible treatment options for breast cancer prevention which may include anastrozole.

All participants wishing to remain on treatment must confirm that they have received a letter informing them of the results before subsequent medication can be dispensed.

Participants who withdraw prematurely during the active treatment period should still continue to be followed up for primary and secondary objective outcomes. Unless the participant specifically withdraws consent for this process, future post-treatment follow-up will continue. Thus, if a participant withdraws after 3 years of active treatment, for example, annual post-treatment follow-up will commence for 7 years (i.e. to 10 years) as described in section 4.5.1.2 and then beyond the 10-year period as described in section 4.5.1.3 (if applicable).

4.5.4 All Adverse Events

4.5.4.1 Adverse Events (AE)

An adverse event is defined as the development of a new, undesirable medical condition or the deterioration of a pre-existing condition following or during exposure to a medicine **whether or not considered causally related to the product.** A medical condition can be a symptom (such as nausea or chest pain) a sign (such as rash or enlarged liver) or an abnormal result of an investigation (including blood tests, x-rays or scans or various types). In clinical studies, an AE can include an undesirable medical condition occurring at any time, even if no study treatment has been administered.

All adverse events will be reported via the relevant follow-up CRF.

4.5.4.2 Serious Adverse Events (SAE)

A serious adverse event is an event that fulfils one or more of the following criteria:

- is fatal
- is life-threatening
- requires in-patient hospitalisation or prolonging hospitalisation
- is a persistent or significant disability or incapacity
- or is an important medical event that may require medical intervention to prevent permanent impairment or damage.

The causality of SAEs (ie, their relationship to study treatment) will be assessed by the PI (or other clinician(s) delegated the role of causality assessment of SAEs), who must answer “yes” or “no” to the question “Is the SAE likely to be caused by one of the IMPs?” on the SAE CRF. If the answer to this question is yes, the SAE will be classified as a Serious Adverse Reaction (SAR) – See Section 4.5.4.3.

SAEs during the treatment period:

SAEs identified as having occurred during the on-treatment period will be reported to the IBIS Central Coordinating Centre within 24 hours. The IBIS-II CCO will report all SAEs to AstraZeneca within 24 hours of receipt from the investigator site.

Pre-planned hospitalisations (i.e. planned prior to randomisation), elective surgery, and pre-existing conditions (unless the condition has worsened) will not be reported as SAEs in the IBIS-II trial. Breast cancer and DCIS should not be reported as an SAE as this signifies an endpoint of the trial. In this case a breast cancer form and final form should be completed.

SAEs during the follow up period:

SAEs which are an event of interest (see section 4.5.1.2), should continue to be collected and reported to the IBIS-II CCO after a patient has completed active treatment i.e. during the annual post-treatment follow-up period by the PI/delegated clinician under expedited timeframes. AEs/SAEs in clinical areas other than those pre-specified will not be actively sought or collected within CRFs during the follow up period and should not be sent to IBIS-II CCO. However, if an investigator becomes aware of a SAR during the follow up period i.e. meets the definition of an SAE and in the investigator's judgement has a causal relationship to a study IMP, this should be reported by the investigator to the IBIS-II team using expedited reporting timeframes, regardless of whether the SAE is a clinical event of interest.

What to report as an SAE in the long term follow up period under expedited conditions:

- ✓ Cerebrovascular event/Stroke/TIA
- ✓ New primary cancers (except basal cell carcinoma, squamous cell carcinoma of the skin and carcinoma in situ of the cervix)
- ✓ Cardiovascular events e.g. angina, myocardial infarction
- ✓ Thromboembolic events e.g. deep vein thrombosis, pulmonary embolism, phlebitis
- ✓ Osteoporosis/Broken/fractured bone
- ✓ Deaths (including underlying cause of death)
- ✓ Any other AE meeting the definition of serious (see above) and in the opinion of the investigator is causally related to the IMP i.e. **all SARs**

What not to report as an SAE in the long term follow up period:

- × New breast cancer or DCIS (endpoint not SAE)
- × AEs that do not meet the definition of serious
- × Pre-planned hospitalisations/elective surgery where the underlying condition does not meet the definition of an SAE
- × Non-protocol specified SAEs (see above) that are not in the opinion of the investigator, related to the IMP

Recording of serious adverse events (open sites)

All serious adverse events will be recorded in the electronic SAE CRF provided, providing the following information as appropriate:

Description of event; dates and times of onset and resolution; event intensity; seriousness; outcome; causality; any action taken (e.g. treatment, diagnostic tests). It is important to distinguish between serious and severe AEs. Severity is a measure of intensity whereas seriousness is defined by the criteria in the previous 4.5.4 sections. An AE of severe intensity need not necessarily be considered serious. For example, nausea, which persists for several hours, may be considered severe nausea, but not a SAE. On the other hand, a stroke resulting in only a limited degree of disability may be considered a mild stroke but would be a SAE.

Additional details on action taken, including diagnostic procedures, will be recorded for any patients with vaginal bleeding or discharge. Any patients who report abnormal vaginal bleeding, or discharge or pelvic pain or pressure should be promptly investigated. It is not mandatory that the code is broken in such cases, but this may be done if requested by the treating physician, when necessary for further treatment

Reporting of serious adverse events

Investigators and other site personnel must inform London IBIS Central Office of all SARs and any SAE in a specified event of interest within 24 hours, of becoming aware of the event.

The electronic SAE CRF will be sent to the IBIS Central Coordinating Centre and a hard copy of the SAE CRF should be printed, signed by the local principal investigator (or other clinician(s) delegated the task of causality assessment of SAEs) and faxed to the IBIS Central Coordinating Centre as soon as possible (Fax: +44 (0)20 7882 3886)

Follow-up information on SAEs must also be reported by the investigator within the same time frame.

If a non-serious AE becomes serious and meets the definition of an SAE for an event of interest (see section 4.5.1.2), this and other relevant follow-up information must also be provided within 24 hours as described above.

4.5.4.3 UK Registry identified SAEs:

Open UK sites

Any data received via registries or Hospital Episode Statistics (HES) in the UK will be assessed by the data management (DM) team as part of a bulk dataset. This long-term follow-up (LTFU) data will be received by a LTFU data manager based at IBIS-II CCO, periodically and at different time points, from a variety of organisations within each of the devolved UK nations. Pseudoanonymised data, recording only the SNO against event details, identifying whether a participant is on active treatment or not, will be tabulated and forwarded to the IBIS-II DM team. The data will be checked, with active participants made a priority over those off study treatment, for incidence of breast cancers, deaths and any other clinical events that could require a SAE report.

It will be cross-checked against existing events recorded for the study. All events newly identified via the passive LTFU data, i.e. not previously reported in the IBIS-II database, will be verified with the IBIS-II sites by the IBIS-II DM team. Where appropriate, sites will be requested to complete all relevant CRFs as fully as possible. If it is unclear from the information provided via the passive LTFU data, whether a newly identified event meets SAE seriousness criteria, a member of the IBIS-II team will request that site staff investigate further. If the patient is 'lost to follow up', the event may be listed as 'unverified'.

If the study site confirms that the clinical event queried meets the seriousness criteria for a SAE, a completed SAE CRF should be sent via the usual method to the IBIS-II CCO, within 24 hours of SAE status confirmation

If the clinical event cannot be confirmed as meeting the seriousness criteria by the site, then further queries to the treating consultant (if applicable) or General Practitioner (GP) may be required. The IBIS-II DM team will make up to 3 attempts to chase further information, after which if no further details can be obtained then a decision on the final action will be made on a case by case basis following a review by the IBIS-II team.

Once the data for participants on treatment has been checked and where applicable, queries have been sent to the relevant sites, the above process will be repeated for participants who are off treatment. As these participants are no longer taking study medication there is a lower safety risk associated with them.

If a clinical event of interest is confirmed as an SAE by a site or a SAR, they should follow the normal timeline and report the event as an SAE within 24 hours of becoming aware of the confirmed SAE. Those that are unable to be verified will be sent to AZ as 'unverified' line listings every six months.

The development of a new **non**-breast primary cancer (including DCIS) which has been identified after inclusion into the clinical trial should be regarded as a serious adverse event. Exceptions to this are basal cell carcinoma, squamous cell carcinomas of the skin and carcinoma in situ of the cervix where diagnosis and treatment of these should be reported as an AE rather than an SAE.

Closed UK sites

There are limitations of registry data collection, in that seriousness and relatedness cannot be assessed by this method of data collection once the sites have closed. A risk adapted approach is now merited as the safety profile of anastrozole is well-established across multiple trials and long follow up periods (as of 31 July 2016, the total cumulative exposure

figure for Arimidex (anastrozole) in the marketed setting is calculated to be in excess of 8 million patient years). Anastrozole has also been recommended by the National Institute of Clinical Excellence (CG164) as a first-line therapy for women at risk of breast cancer without osteoporosis. All participants are now off-treatment and the half-lives of anastrozole is relatively short (50 hours), therefore collection of follow up data from the digital registries poses a minimal risk to participants.

All data received via registries in the UK will be assessed by the data management (DM) team as part of a bulk dataset. This long-term follow-up data will be received by an independent data manager on a yearly basis from the various devolved UK data registries and Public Health England as previously described. The data will be screened for deaths, new and recurrent breast cancers and other ICD-coded events listed in Table 1. All newly identified events that meet the criteria listed in 4.5.1.2 will be sent to AZ as an ‘unverified’ line listing once a year. This will provide data on AEs that should be reported in the long term follow up period under expedited conditions but with the limitations discussed above.

4.5.4.4 Suspected Unexpected Serious Adverse Reaction (SUSAR) and Serious, Suspected Adverse Reactions (SSARs)

Local centre investigators are responsible for assessing seriousness and relatedness of an adverse reaction.

It will be the responsibility of AstraZeneca to confirm whether any reported SARs are a Suspected Unexpected Serious Adverse Reaction (SUSAR) or a Serious Suspected Adverse Reaction (SSAR). AstraZeneca will forward any SUSARs to the regulatory authority. (Appendix 3a and 3b)

SUSARs identified from UK registry data post UK site closures

It will be noted in the DSUR report that seriousness and relatedness cannot be determined from the digital registries.

If a SUSAR is identified by AZ from the registry data, then this will be reported as such. However, the usual timelines for reporting a SUSAR cannot be adhered to with registry data events.

See Appendix 3a and 3b for a summary of safety reporting procedures

5. Toxicity and Hazards

Anastrozole has been used in the treatment of breast cancer since 1995 and is generally well tolerated. Reported adverse events have usually been mild to moderate with only a few withdrawals from treatment due to undesirable events. These include hot flushes, vaginal dryness and hair thinning. Anastrozole may also be associated with gastrointestinal disturbances (anorexia, nausea, vomiting and diarrhoea), asthenia, arthralgia, somnolence, headache or rash.

Anastrozole has been used in advanced breast cancer and has been found to be at least as effective as tamoxifen, but with fewer side effects (Bonneterre *et al*., 2000) (see Table 5). Until recently its use as an adjuvant in early breast cancer has been restricted to clinical trials, but it has now been licensed for use in the adjuvant setting in several countries.

As all women will be postmenopausal, pregnancy risk will not be an issue in this trial.

Results from an adjuvant trial (ATAC) (ATAC Trialists, 2002) enrolling over 9000 postmenopausal patients indicate that anastrozole is more effective in reducing early recurrences than tamoxifen (P=0.013). At the time of this report, the median follow-up was 33.3 months and median duration of treatment was 30.7 months. The side effect profile is also generally more favourable (Table 5), with fewer endometrial cancers, thromboembolic events, strokes, and hot flushes. However, musculoskeletal symptoms (mostly arthralgia) and fractures of osteoporotic sites and other sites are increased. It is not yet clear the extent to which bone loss associated with anastrozole. Also no studies to date have been conducted to investigate concomitant use of vitamin D and calcium or bisphosphonate in

combination with anastrozole. Because of the profound reduction of circulating oestrogen levels in women taking anastrozole, effective management of potential bone loss is likely to be important. Detailed bone density studies are currently underway in the adjuvant trials, and further studies will also be undertaken within this trial. A separate bone sub-study will address these issues in the prevention stratum.

Table 5. Pre-specified adverse events in the ATAC adjuvant trial (%)

	Anastrozole (N=3125)	Tamoxifen (N=3116)	Combination (N=3125)
Hot flushes	34.3	39.7	40.2
Nausea and vomiting	10.5	10.2	11.7
Fatigue/Tiredness (Asthenia)	15.7	15.1	14.0
Mood disturbances	15.5	15.2	15.6
Musculo-skeletal disorders	27.8	21.2	22.1
Vaginal bleeding	4.5	8.1	7.7
Vaginal discharge	2.8	11.4	11.4
Endometrial cancer[1]	0.1	0.5	0.2
Fractures	5.8	3.7	4.6
Fractures of spine/hip/wrist	2.2	1.4	1.6
Ischaemic cardiovascular disease	2.5	1.9	2.2
Ischaemic cerebrovascular event	1.0	2.1	1.6
Venous thromboembolic events	2.1	3.5	4.0
Deep venous thromboembolic events	1.0	1.7	2.0
Cataracts	3.5	3.8	3.4

[1] Excluding patients with hysterectomy at baseline

Anastrozole is not recommended for use in premenopausal women as safety and efficacy have not been established in this group of women. Anastrozole has not been investigated in patients with severe hepatic or severe renal impairment, but women with these conditions

will not be eligible for the trial. The potential risk/benefit to such patients should be carefully considered before administration of anastrozole.

Antipyrine, warfarin and cimetidine clinical interaction studies indicate that the co-administration of anastrozole with other drugs is unlikely to result in clinically significant drug interactions mediated by cytochrome P450.

Hepatic changes (elevated gamma-GT or less commonly alkaline phosphatase) have been reported in patients with advanced breast cancer, many of whom had liver and/or bone metastases. A causal relationship for these changes has not been established. Slight increases in total cholesterol have also been observed in clinical trials with anastrozole.

Daily doses of anastrozole up to 10 mg do not have any effect on cortisol or aldosterone secretion, measured before or after standard ACTH challenge testing. Corticoid supplements are therefore not needed.

Events of Carpal Tunnel Syndrome have been reported in patients receiving anastrozole treatment in clinical trials in greater numbers than those receiving treatment with tamoxifen. However, the majority of these events occurred in patients with identifiable risk factors for the development of the condition.

In the ATAC trial, ischaemic cardiovascular events were reported more frequently in patients treated with anastrozole compared to those treated with tamoxifen, although the difference was not statistically significant. The observed difference was mainly due to more reports of angina pectoris and was associated with a sub-group of patients with pre-existing ischaemic heart disease.

Sensory disturbances (including paraesthesia, taste loss and taste perversion) have been reported more frequently in patients receiving anastrozole than those on tamoxifen in clinical trials. In order to ensure that participants still on treatment are aware of this

newly listed side effect (added to Arimidex Summary of Product Characteristics [SmPC] v160114), they must be informed of this at their next clinic appointment.

The full list of adverse reactions to anastrozole is available as a separate document. (Astra Zeneca Core Data sheet)

6. Analysis of Data and Statistical Considerations

6.1 Randomisation

Analysis will be based on randomised treatment option (intent to treat or ITT). For this reason, it is important to attempt to ensure good compliance before randomisation.

Randomisation will be stratified by individual major (hub) centre only. The randomisation will not be stratified for the other risk factors, but they will be used to achieve retrospective stratification at the time of analysis.

6.2 Endpoints

The primary endpoint is the development of locally histologically confirmed breast cancer, both invasive and non-invasive (i.e. including DCIS). The principal analysis for comparing the active treatment to placebo will be performed when 117 cases of breast cancer have been diagnosed in the prevention stratum (see section 6.3 for more details). It is anticipated that this will occur after a median follow-up of 5 years. Primary endpoint data will be collected from participating women until the end of trial definition (LPLT + 5 years of follow up).

Adherence to treatment protocol will cease if a woman develops breast cancer and a breast cancer CRF should be returned. All breast cancer pathology must be reviewed by a pathologist approved by the UK National Breast Screening Programme or foreign equivalent. In addition, a tissue block or where unavailable 20 sections. will be required in consenting women for all breast tumours developing after entry into the trial.

It is recognised that breast cancer mortality is an important exploratory endpoint and this will also be analysed. However, the power to detect this within 10 years is marginal. Continued long-term follow-up beyond 10 years, as well as an overview of other similar trials will be needed to obtain clear results on this question. Total and cause-specific mortality will be analysed and the Final CRF (if still on active treatment) and Death CRF should be returned for any woman who dies of any cause while on therapy.

All women will be followed up for cancer incidence (breast and other cancers) and cause of death using available NHS registries as described in section 4.5.1.4. During the 5-year period of active follow-up and the post-treatment follow-up period serious medical conditions will be recorded including myocardial infarction and other heart problems, thromboembolic events (superficial and deep), cardiovascular events, osteoporosis, fractures, and other cancers.

Basic information on prescriptions and hospital visits during the 5 years of active treatment for each participant will also be recorded.

6.3 Numbers of Volunteers and Power

The sample size for the prevention stratum will be a minimum of 3500 (although target will be 4000 to allow for possible changes in subsequent patient characteristics).

Three factors determine the numbers needed for this study. They are (i) the absolute risk level of the participants, (ii) the size of the smallest reduction thought to be worthwhile and (iii) compliance rates. Initially assumptions were based on an incidence of 6 cases of breast cancer per 1,000 women per year for high-risk women (as in IBIS-I), and a compliance adjusted breast cancer reduction of 50% for anastrozole. However interim figures indicate that incidence is much higher. The overall event rate is 6.6 cases of breast cancer per 1000 women per year which, with a 50% reduction in the anastrozole arm, would translate to 9 cases of breast cancer per 1000 women per year for placebo. Thus the expected number of new cancers in each arm after 5 years of median

follow-up for a total trial size of 3,500 is 78 for placebo and 39 for anastrozole leading to an expected chi square of 13 and power in excess of 90% for a 5% significance level.

Long term follow-up Volunteers and Power

Participants from three participating countries will be included in the long-term follow up study, based on good data return during the active clinical trial, number of endpoints, and number of participants recruited. For a 60% reduction with anastrozole compared to placebo, 2584 participants and 208 events over the next 10 years would be needed to achieve a power of 90% (alpha=0.05) (superiority).

6.4 Data Monitoring

Monitoring of the data will be undertaken every 12 months and summary analyses will be provided to the independent members of the Trial Steering Committee. No formal interim analyses are planned. This Committee will meet at least every 12 months and will have the responsibility to recommend whether to stop the study early. As a general guideline, a difference between arms should exceed three standard deviations for some mortality endpoint before a decision to stop prematurely is taken, although other factors will also have to be taken into consideration. The Steering Committee will be responsible for the final decisions on such issues.

6.5 Unblinding the Randomisation

The study is a double blind, placebo controlled, randomised trial i.e. neither the doctor nor the woman will know who is on active treatment. However, should a participant decide to request a codebreak and is on anastrozole, she will be able to continue the remainder of the trial treatment unblinded. Should a participant decide to request a codebreak and is on placebo, she will no longer receive trial medication and will move to the post-treatment phase of the trial.

The treatment code should only be broken in the following circumstances:

1) Where the woman develops breast cancer
2) When a clinician considers there to be valid medical or safety reasons to break the blind
3) When a participant requests unblinding

The clinician must have a legitimate reason to break the blind such as when the study drug is likely to have a significant effect on the clinical management of the subject e.g. in the case of a severe adverse event where it is necessary for the clinician to know which treatment the subject is receiving before the subject can be treated.

A written request, detailing the reason for the code break and signed by the PI, should then be faxed to the national coordinating office. The request will then be passed to the central coordinating office where once approved, the code will be broken by the IBIS-II programmer and faxed back to the PI.

Where required by designated national sites (due to time differences), access to the information for code breaks can be made available via a secure website.

The treatment code is located on a secure server at the IBIS-II CCO. There is no intention to break the code at the end of the trial.

6.6. End of Trial

The end of trial is defined as Last Patient Last Visit (LPLV). This will occur when the last patient completes both 5 years of active trial treatment + 5 years' annual post-treatment follow-up (10 years in total). Follow-up beyond 10 years, applicable to those participants that have reached this stage, should be considered as an observational element to the study. This will ensure continued annual follow-up of all participants until LPLV (2022).

7. Consent and Ethics Committee Approval

The study has been approved by the North West Multi-centre Research Ethics Committee (MREC). The study will also require local approval at participating centres.

This study involves evaluation of medicines for an unlicensed indication and is being carried out under EudraCT no. 2004-003991-12. Non-negligent indemnity for women joining the study will be provided worldwide by Queen Mary's University of London.

All women interested in participating in the study will receive a patient information sheet (PIS) that explains the purpose of the study and warns of the potential short term and long-term side effects of anastrozole. The clinician (Principal Investigator (PI) or other persons delegated to take consent) will clarify and discuss any points arising from the information literature. Participants will then be required to sign an informed consent form (CF) prior to undergoing any tests. Consent may be taken by the research nurse if the PI has approved this delegation. In such circumstances the PI should be available if necessary, so that the participant may then re-discuss the study with the PI, who will then counter-sign the CF. Further information and counselling will be made available throughout the study in response to a woman's request or following significant new information on the side effects of anastrozole. This will be provided by the clinician or the research nurse appointed for this study. All women will be free to stop treatment at any stage, and it will be made clear that leaving the study will not prejudice any future management which may be needed.

Women will be asked to consent

i) to join the study,
ii) to have their past and future medical records, mammograms, etc. examined, including those in a cancer registry or death certificate,
iii) to the storage of personal information for the purposes of the study,
iv) for a blood sample at entry and on follow-up for risk factor biomarkers and study of genes related to disease risk,
v) to access to any pathology specimen for anonymous testing for factors related to disease or side effects,
vi) to receive additional questionnaires from time to time to obtain further relevant information.

All research tests involving DNA or immunohistochemistry will be performed blindly on a case-control basis and results will not be linked to individuals. Thus, it will not be possible to inform women of the results of any tests because of the anonymous nature of all testing.

Participants have been informed of PID data collection and continued storage for the purposes of long term follow up. To clarify this, the process was reviewed and approved by the National Information Governance Board for Health and Social Care (ECC 6-03(FT8)2012), now known as section 251 support from the Confidential Advisory Group.

8. References

ATAC Trialists' Group. (2002). Anastrozole alone or in combination with tamoxifen versus tamoxifen alone for adjuvant treatment of postmenopausal women with early breast cancer: first results of the ATAC randomised trial. *Lancet*, **359**: 2131-2139.

ATAC Trialists' Group. (2005). Results of the ATAC (Arimidex, Tamoxifen, Alone or in Combination) trial after completion of 5 years' adjuvant treatment for breast cancer. *Lancet*, **365**: 60-62.

ATAC Trialists' Group (2008) Effect of anastrozole and tamoxifen as adjuvant treatment for early-stage breast cancer: 100-month analysis of the ATAC Trial' *Lancet Oncol,* **9:** 45-53

Beral V, Million Women Study Collaborators. (2003). Breast cancer and hormone-replacement therapy in the Million Women Study. *Lancet*, **362**: 419-427.

Bonneterre J, Buzdar A, *et al.* (2001). Anastrozole is superior to tamoxifen as first-line therapy in hormone receptor-positive advanced breast carcinoma. *Cancer*; **92(9):** 2247-2258.

Buzdar AU, Jonat W, Howell A, Plourde PW. (1997). Arimidex: a potent and selective aromatase inhibitor for the treatment of advanced breast cancer. *J. Steroid Biochem. Molec. Biol.*, **61:** 145-149.

Buzdar AU, Jonat W, Howell A, Jones SE, Blomqvist CP, Vogel CL, Eiermann W, Wolter JM, Steinberg M, Webster A, Lee D. (1998). Anastrozole versus megestrol acetate in the treatment of postmenopausal women with advanced breast carcinoma: results of a survival update based on a combined analysis of data from two mature phase III trials. *Cancer*; **83:** 1142-1152.

Buzdar A, Bonneterre J, Nabholtz JM, *et al*. (2000). Anastrozole is superior to tamoxifen as first-line therapy for advanced breast cancer (ABC) in postmenopausal (PM) women. Findings highlight the importance of receptor status assessment prior to treatment initiation. *Ann. Oncol.,* **11:** 25-25 Suppl. 4.

Cummings SR, Eckert S, Krueger KA, *et al*. (1999). The effect of raloxifene on risk of breast cancer in postmenopausal women. *JAMA,* **281:** 2189-2197.

Cuzick J. (2000). A brief review of the breast cancer prevention trials. *Eur. J. Cancer*, **36:** S49-S56.

Cuzick J. *et al.* (2002). First Results from the IBIS-I Breast Cancer Prevention Trial. *Lancet*, **360**: 817-824.

Cuzick J. *et al.* (2003). Overview of the Main Outcomes in Breast Cancer Prevention Trials. *Lancet*, **361**: 296-300.

Cuzick J. *et al* (2007). Long-Term Results of Tamoxifen Prophylaxis for Breast Cancer — 96-Month Follow-up of the Randomized IBIS-I Trial **J Natl Cancer Inst**, 99: 272-82

Cuzick J. *et al.* (2014). Anastrozole for prevention of breast cancer in high-risk postmenopausal women (IBIS-II): an international, double-blind, randomised placebo-controlled trial. *The Lancet,* 383, Issue: 1041 – 1048.

EBCTCG-Early Breast Cancer Trialists' Collaborative Group. (1992). Systemic treatment of early breast cancer by hormonal, cytotoxic, or immune therapy. *Lancet*; **339:** 1-15, 71-85.

EBCTCG-Early Breast Cancer Trialists' Collaborative Group. (1998). Tamoxifen for early breast cancer: an overview of the randomised trials. *Lancet*; **351:** 1451-1467.

EPOS –The European Prospective Osteoporosis Study (Epos) Group. (2002). Incidence of Vertebral Fracture in Europe: Results from the European Prospective Osteoporosis Study (EPOS). *J. Bone Min. Res.*, **17:** 716-724.

Fallowfield L, Fleissig A, Edwards R, West A, Powles TJ, Howell A, Cuzick J. (2001). Tamoxifen for the prevention of breast cancer: psychosocial impact on women participating in two randomized controlled trials. *J. Clin. Oncol.*, **19**: 1885-1892.

Fisher B, Costantino J, Redmond C, *et al*. (1989). A randomised clinical trial evaluating tamoxifen in the treatment of patients with node negative breast cancer who have oestrogen receptor positive tumors. *N. Engl. J. Med.,* **320:** 479-484.

Fisher B, Costantino JP, Wickerham DL, *et al*. (1998). Tamoxifen for prevention of breast cancer: Report of the National Surgical Adjuvant Breast and Bowel Project P-1 Study. *JNCI*, **90:** 1371-1387.

Fornander T, Cedermark B, Mattsson A. *et al.* (1989). Adjuvant tamoxifen in early breast cancer: occurrence of new primary cancers. *Lancet*; **i:** 117-120.

Geisler J, King N, Dowsett M, *et al*. (1996). Influence of anastrozole (Arimidex), a selective, non-steroidal aromatase inhibitor, on *in vivo* aromatisation and plasma oestrogen levels in postmenopausal women with breast cancer. *Br. J. Cancer*; **74:** 1286-1291.

Houghton J, George WD, Cuzick J, Duggan C, Fentiman IS, Spittle M; UK Coordinating Committee on Cancer Research; Ductal Carcinoma in situ Working Party; DCIS trialists in the UK, Australia, and New Zealand. (2003). Radiotherapy and tamoxifen in women with completely excised ductal carcinoma in situ of the breast in the UK, Australia, and New Zealand: randomised controlled trial. *Lancet,* **362(9378)**: 95-102.

Jackson IM, Litherland S & Wakeling AE. (1991). Tamoxifen and other antioestrogens. In *Medical Management of Breast Cance*r, eds: Powles, T.J. & Smith, I.E. Publ. Martin Dunitz, London.

Jakesz R, Hausmaninger H, Samonigg H, *et al*. (1999). Comparison of adjuvant therapy with tamoxifen and goserein vs CMF in premenopausal stage I and II hormone-responsive breast cancer patients: Four-year results of Austrian Breast Cancer Study Group (ABSCG) Trial 5. *Eur. J. Cancer,* **35:** S83-S83, Suppl.4.

Jonat W, Howell A, Blomqvist C, *et al.* on behalf of the anastrozole Study Group. (1996). A randomised trial comparing two doses of the new selective aromatase inhibitor anastrozole with megestrol acetate in postmenopausal women with advanced breast cancer. *Eur. J. Cancer*; **32A:** 404-412.

Nabholtz JM, Buzdar A, Pollacj M, *et al*. (2000). Anastrozole is superior to tamoxifen as first-line therapy for advanced breast cancer in postmenopausal women – Results of a North American Multicenter Randomized Trial. *J. Clin. Oncol.,* **18:** 3758-3776.

Peto R, Boreham J, Clarke M, Davies C, Beral V. (2000). UK and USA breast cancer deaths down 25% in year 2000 at ages 20-69 years. *Lancet*; **355:** 1822-1823.

Plourde PV, Dyroff M, Dowsett M, *et al*. (1995). Anastrozole: A new oral, once-a-day aromatase inhibitor. *Journal of Steroid Biochemistry*; **53:** 175-179.

Powles T, Eeles R, Ashley S, *et al*. (1998). Interim analysis of the incidence of breast cancer in the Royal Marsden Hospital tamoxifen randomised chemoprevention trial. *Lancet,* **352**: 98-101.

Powles TJ, Tillyer CR, Jones AL, *et al*. (1990). Prevention of breast cancer with tamoxifen – an update on the Royal Marsden Hospital pilot programme. *Eur. J. Cancer,* **26:** 680-684.

Rutqvist LE. (1999). Zoladex and tamoxifen as adjuvant therapy in premenopausal breast cancer: a randomised trial by the Cancer Research Campaign (CRC) Breast Cancer Trials Group, the Stockholm Breast Cancer Study Group, the South-East Sweden Breast Cancer Group and the Gruppo Interdisciplinare Valutazione Interventi in Oncologia (GIVIO). *Proc. ASCO,* **18:** 67a.

Stuart-Harris RC & Smith IE. (1984). Aminoglutethimide in the treatment of advanced breast cancer. *Acta Oncology*; **27:** 721-728.

Sunderland MC & Osborne CK. (1991). Tamoxifen in premenopausal patients with metastatic breast cancer: a review. *J. Clin. Oncol.* 1991, **9:** 1283-1297.

Veronesi U, Maisonneuve P, Sacchini V, *et al*. (2002). Tamoxifen for breast cancer among hysterectomised women. *Lancet,* **359**: 1122-1124.

Wells SA, Santen RJ, Lipton A, *et al.* (1978). Medical adrenalectomy with aminoglutethimide: clinical studies in postmenopausal patients with metastatic breast carcinoma. *Annals Surgery*, **187:** 475-487.

9. Appendix

Appendix 1 – Breast **Cancer Prevention Trials**

Trial (entry dates)	Population (size)	Agent (vs Placebo)	Intended Duration of Treatment
Royal Marsden (1986-1996)	Increased Risk Family History (2,471)	Tamoxifen 20mg	5-8y
NSABP-P1 (1992-1998)	1.6% 5y risk (13,388)	Tamoxifen 20mg	5y
Italian (1992-1997)	Normal Risk Hysterectomy (5408)	Tamoxifen 20mg	5y
IBIS-I (1992-2000)	>2-fold risk (7,139)	Tamoxifen 20mg	5y
MORE (1994-1998)	Normal Risk Osteoporotic Postmenopausal (7705)	Raloxifene 60mg Or Raloxifene 120mg	3y

Appendix 1 continued

Summary Results of Prevention Trials

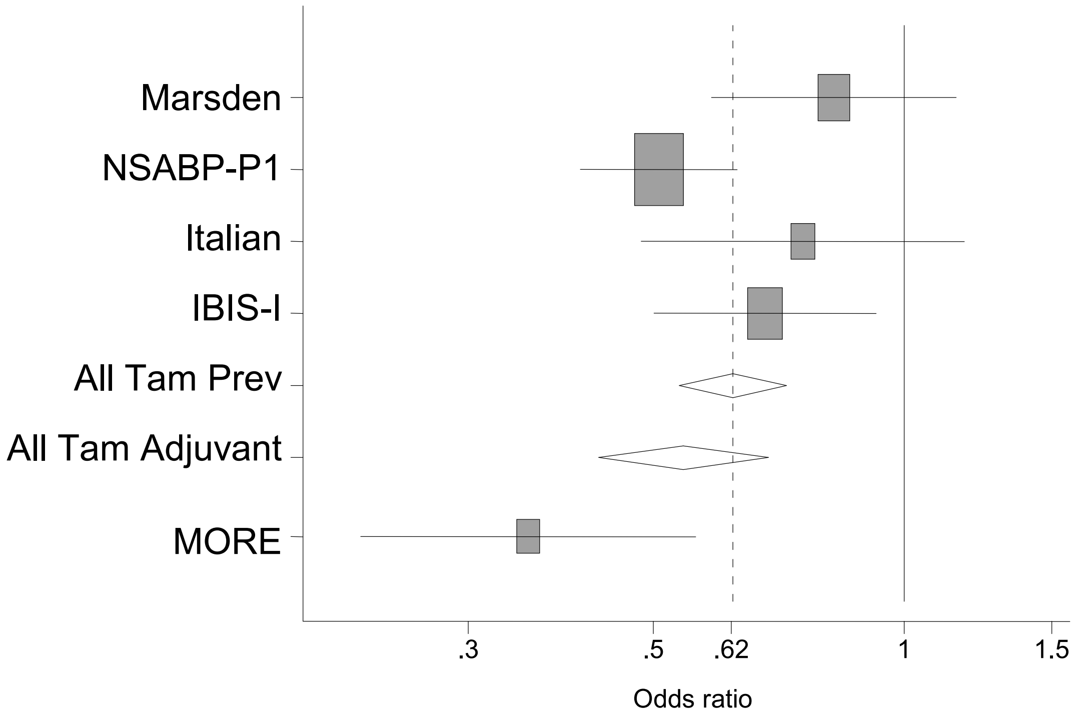

Forest plot of estimates of breast cancer incidence reduction for the prevention trials. The summary estimate for all the tamoxifen trials is OR = 0.62 (95% CI (0.54-0.72)) P<0.001. Test of heterogeneity for all tamoxifen trials gives P=0.09. However, the results of the MORE trial are clearly different (Cuzick et al 2003).

Appendix 2. Summary of Drug Handling Guidelines

Astra Zeneca Responsibilities:

The drug handling company IPS(A) will supply to the IBIS London Office. The two formulations used in the trial will be packaged as bulk primary packs, in boxes of 100, and labelled with batch numbers and expiry dates of the product. Each primary pack will contain 224 tablets in an HDPE (High Density Poly ethylene) bottle.

The Active and Placebo Anastrozole will be supplied in Small 75ml HDPE Bottles. Bottles will be capped with tamper evident child resistant lids.

Drug supplies for the study will be stored below 30˚C, with the placebo formulation separated from the active formulation.

IBIS Central Coordinating Centre Responsibilities:

These are fully described in the Investigational Product Ordering, Labelling, Supplying, Handling & Storage for IBIS-II Clinical Trial Standard Operating Procedure (SOP).

Appendix 3a. SAE reporting flow chart – open sites

Flow diagram 1 Reporting of a SAR or Serious Adverse Event (SAE) for a clinical event of interest by CCO to relevant authorities during follow up (i.e. questionnaire follow up)

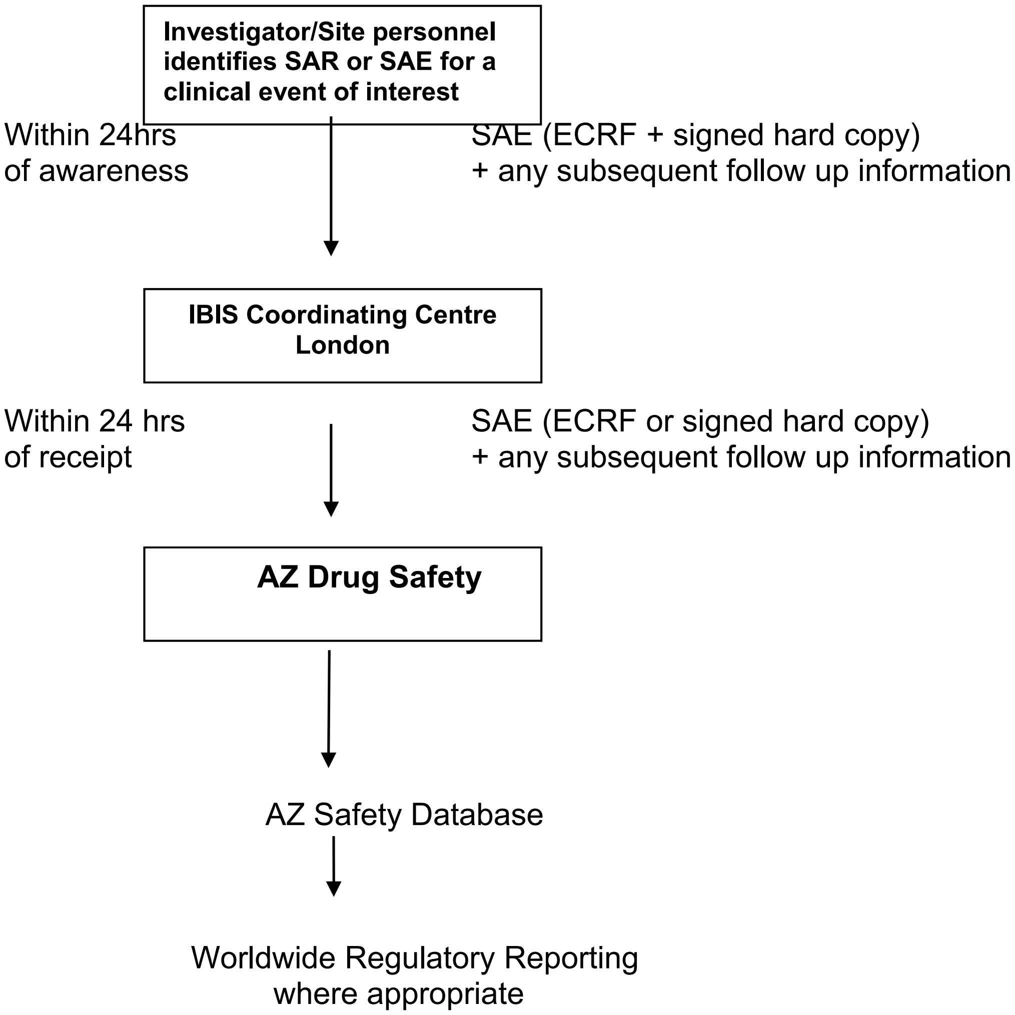

Follow up information on a SAR/SAE in clinical event of interest must be reported by the investigator to the CCO in 24 hours and by CCO to AZ in the same time frame as the initial report. i.e. 24 hours since receipt from investigator. 24 hour deadline refers to a timeline within the normal working hour schedule.
AZ may request specific follow up information which will be sent to the Investigator via the CCO.   SAEs that are not related (i.e. not a SAR) or not relating to clinical event of interest do not have to be reported during the questionnaire follow up period.

For further details, please refer to the 'SAE Reporting - Investigator Responsibilities' SOP and the 'Operational Guide' SOP

Appendix 3b. SAE reporting flow chart – registry data

Reporting of clinical event of interest identified from digital registries (only once on-site data has stopped) by CCO to relevant authorities

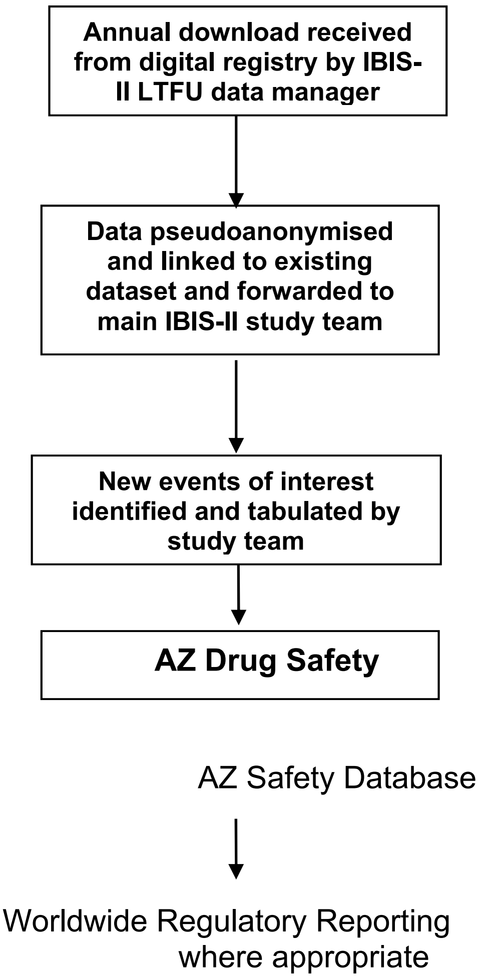

For further details, please refer to the 'Operational Guide' SOP
