# Supplementary material for: The impact of body mass index on breast cancer incidence among women at increased risk: an observational study from the International Breast Intervention Studies
Source: Breast Cancer Res Treat. 2021 Mar 3;188(1):215–23. doi: 10.1007/s10549-021-06141-7 (PMC8233270; doi:10.1007/s10549-021-06141-7)
Supplement: Supplementary file 2 — (PDF 162 KB) [file 10549_2021_6141_MOESM2_ESM.pdf]

PROTOCOL

An international multi-centre study

of

tamoxifen vs placebo

in

women

at

increased risk of breast cancer

May 2001

Table of Contents

Table of Contents	Page Number
**1 Introduction**	1
1.1 Aims	1
1.2. Background	1
1.3. Pre-clinical basis for use of an 'anti-oestrogen'	3
**2 Study Design**	4
2.1 Outline	4
2.2 Dose and Duration	4
2.3. Participating Centres and Recruitment	4
2.4. Eligibility	5
2.5. Exclusions	6
2.6 Hormone Replacement Therapy	7
2.7 Randomisation	7
2.8 Investigations	7
2.8.1 Mammography	7
2.8.2 Personal and medical details	7
2.8.3 Blood samples	8
2.8.4 Special Investigations	8
2.9 Follow up	8
**3 Data Analysis**	9
3.1. Randomisation	9
3.2. Endpoints	10
3.3 Number of Volunteers and Power	10
3.4 Data Monitoring	11
**4 Toxicity and Hazards**	11
4.1. Unblinding the Randomisation	13
4.2. Adverse Events	13
**5 Consent and Ethics Committee Approval**	13
**6 References**	15
**7 Appendices**	
7.1.Entry Criteria	22
7.2 Revised Power Calculations	23
7.3 Recruitment Update	24
7.4 Pregnancy Protocol	25

International Breast Cancer Intervention Study

A Multicentre Trial of Tamoxifen to Prevent Breast Cancer

Working Party Chairman	Dr. Jack Cuzick, 020 7269 3162
Clinical Advisor	Trevor Powles, 020 8642 6011, ext 3363
Senior Study Co-ordinator	Clare O'Neill
	IBIS<br>P.O.Box 123<br>Lincoln's Inn Fields<br>London WC2A 3PX<br>Tel 020 7269 3162
	**RANDOMISATION NUMBERS TEL: 020 7269 3162**<br>**FAX: 020 7269 3429**
Blood Samples	Bill Richmond<br>Diagnostic Chemical Pathology<br>St. Mary's Hospital<br>Praed Street<br>London W2 1NY<br>Tel: 020 7725 1374

Working Party

Dr E. Anderson
Professor M. Baum
Professor R. Blamey
Dr. J. Cuzick (Chairman)
Ms. Sylvia Denton
Dr. L Fallowfield
Professor I. Fentiman
Professor J. Forbes
Professor D. George
Professor Dr. A. Goldhirsch
Dr. A. Howell
Dr. A-R Henry
Dr. K. Holli
Professor C. Normand
Dr. T. Powles
Dr. B. Richmond
Dr. Torner-Garcia

The study is conducted as a collaborative project of the Breast Cancer Trials Co-ordinating Subcommittee (chairman: Dr. A. Howell) of the United Kingdom Co-ordinating Committee on Cancer Research.

1 Introduction

1.1 Aims

A study to evaluate the reduction in incidence of, and mortality from, breast cancer associated with taking tamoxifen daily for five years. The study will involve analysis of many endpoints to provide a risk-benefit assessment of this intervention.

1.2 Background

Each year in the UK, there are about 25,500 new cases of breast cancer and approximately 15,000 women die from the disease. It is the most common female cancer and is responsible for about 20% of the deaths from cancer in women. Many women undergo mutilating surgery with subsequent chemotherapy or radiotherapy, but there has been little improvement in survival. If adjuvant chemo- or hormone therapy was used more widely, the survival figures might be improved by as much as 20-30%. Full implementation of the national screening programme could also lead to similar reductions in mortality. However, neither of these approaches will affect incidence and it is clear that with currently available treatments, the prospects for making a major impact on the morbidity and mortality from breast cancer lie more in the area of prevention. The national screening programme does however offer the opportunity to identify women at high risk of breast cancer and is very complementary to the aims of a prevention trial. The screening programme could provide for the routine identification of high risk women and special provisions are already being made in some places to begin screening at a younger age for women with a strong family history of breast cancer.

The success of the oestrogen agonist tamoxifen in preventing recurrence and reducing mortality in established disease, (EBCTCG, 1992), its apparent ability to prevent or delay a substantial number of new contralateral tumours (Cuzick & Baum, 1985; Fornander *et al.*, 1989; Fisher *et al.*, 1989) and its very low toxicity makes it an attractive agent to consider in a preventive setting. Data collected by Zeneca on new contralateral breast cancers has been reported by Nayfield *et al.* (1991) and is reproduced below:

Cumulative frequency of contralateral breast cancers in clinical trials of adjuvant tamoxifen therapy compared with placebo or observation in women with early-stage breast cancer				
	Tamoxifen-treated patients		Controls	
Clinical trial	No. of patients	No. of cancers	No. of patients	No. of cancers
NATO	564	15	567	17
Scottish	661	9	651	12
Stockholm	931	18	915	32
Copenhagen	164	3	153	4
Toronto-Edmonton	198	3	202	3
ECOG 1178	91	1	90	3
NSABP B-14	1419	23	1428	32
CRC	947	7	965	18
Total	4975	79 (1.6%)	4971	121 (2.4%)

In summary, there were 121 new contralateral breast cancers in the control group and 79 in the tamoxifen-treated group, giving a 33% reduction. Data from the overview of a larger group of trials (EBCTCG, 1992) showed a 39% reduction in contralateral breast cancer.

A pilot study (Powles *et al.*, 1989, 1990) has demonstrated the feasibility of giving tamoxifen to well women and has confirmed the low frequency of short-term side-effects in such a setting. Compliance of over 75% has been achieved in this study. The use of tamoxifen in benign breast conditions has also been well-tolerated (Fentiman & Powles, 1987; Fentiman, 1989).

However, the possibility of some long term adverse effect of tamoxifen needs to be considered. Originally, because of the anticipated anti-oestrogen properties of tamoxifen, the major concerns related to the possibility of accelerating osteoporosis or increasing heart disease. The evidence thus far on these issues has been encouraging. Studies by Fentiman *et al.* (1988), Powles *et al.* (1989, 1990) and Turken *et al.* (1989) have shown no loss of bone mineral content in women given tamoxifen, and several studies (Rossner & Wallgren, 1984; Caleffi *et al.*, 1988; Bruning *et al.*, 1988; Powles *et al.*, 1989, 1990; Love *et al.*, 1990) indicate that tamoxifen has little effect on HDL-cholesterol but reduces LDL-cholesterol levels by about 15-20% suggesting that tamoxifen might substantially reduce the risk of heart disease in postmenopausal women. In the Scottish

adjuvant trial, where tamoxifen was given to the women for 5 years, there was a highly significant reduction in mortality from myocardial infarction (McDonald and Stewart, 1991). Recently the Stockholm trial has also shown a reduction in heart disease which was greater for women taking 5 years of tamoxifen than for those only treated for 2 years (Rutqvist & Mattson, 1993).

Other potential side effects include the possibility of an increased incidence of other cancers, especially of the endometrium, an increase in thromboembolic events, and visual disturbances. These and other risks are discussed in section 4: Toxicity and Hazards.

The human evidence of tamoxifen's value in established breast cancer is the best reason for considering its use in prevention. However, supporting evidence also exists from animal experiments, and its cytostatic activity in these systems suggests its benefits in humans might be greater in a preventive setting than in the adjuvant context. These arguments are made in some detail in Cuzick *et al.* (1986) and Fentiman (1989) and are briefly recounted below.

1.3 Pre-clinical basis for use of an "anti-oestrogen"

Experimental evidence leaves little doubt that oestrogen is involved in carcinogenesis of murine mammary tumours. Both the DMBA and NMU induced mouse mammary tumours require intact ovarian function for their development. Ovariectomy will prevent tumour development but normal tumour development is restored by oestrogen administration even after a delay of several weeks. Ovarian stimulated or oestrogen stimulated tumour development is prevented by concomitant tamoxifen administration, (Jordan *et al.*, 1980; Jordan, 1981). These results seem to indicate that chemically induced tumours require oestrogenic promotion in order to complete the carcinogenic process.

Epidemiological evidence also supports the hypothesis that ovarian function is required to promote naturally induced breast cancer (Miller & Bulbrook, 1980). For example early menarche or naturally occurring late menopause are both associated with an increased risk of breast cancer (Kelsey, 1979). Also, the incidence of breast cancer is substantially reduced by oophorectomy or radiation-induced menopause, (MacMahon & Feinlieb, 1960; Hirayama & Wynder, 1962) and the degree of protection is related directly to the age of artificial menopause (MacMahon & Feinlieb, 1960).

2 Study Design

2.1 Outline

A multicentre, randomised clinical trial of 7,000 women aged between 45 and 70 years who have a risk of breast cancer at least twice that of the general population is proposed. Women aged 40-44 with a 4-fold elevation of breast cancer risk and those aged 35-39 with a 10-fold elevation of risk are also eligible. The specific categories for eligibility are shown in Section 2.4. In general terms increased risk is determined from family history, previous lobular carcinoma in situ or atypical hyperplasia. All women will have a mammogram taken to exclude breast cancer before randomisation and a blood sample will also be taken for cholesterol analysis.

2.2 Dose and Duration

Women will be randomised to receive either tamoxifen 20 mg/day for 5 years or placebo. Tablets will be supplied in numbered light-proof containers containing a 6 month supply. Each container will have extra tablets to allow for some delay in the next appointment. **Physicians must attempt to determine that compliance is likely to be good before randomising subjects.**

2.3 Participating Centres and Recruitment

UK

Each centre will be expected to recruit an agreed number of new participants per year to reach their target over 3 years.

Initially recruitment may be from patients already known to the breast clinics at each local centre and also from women who have volunteered to take part in the study following press publicity. They are recommended to discuss the study with their GP. In some centres there will also be referrals from the national screening programme based on family history. However, this will only be done in centres where a good liaison exists between the screening centre and the breast clinic, and where an invitation to join the study will not prejudice participation in the screening programme. The local co-ordinators will also publicise the study to interested GPs.

Abroad

Additional centres are also being planned in Ireland, Germany and Spain. International Breast Cancer Study Group (IBCSG) centres in Australia, New Zealand, Switzerland and elsewhere will also participate in the study and this will be co-ordinated through the IBCSG. Where possible (Australia and New Zealand excepted) all foreign centres will randomise through the central office in London, but data collection and cholesterol assays will be carried out nationally. Data forms and blood sample aliquots will be transferred to the central office on a regular basis.

2.4 Eligibility

a) Women must satisfy at least one of the entry criteria listed below.

b) A mammogram must have been taken within the last year indicating no malignant disease.

c) A signed consent form must have been obtained.

Entry Criteria

The entry criteria are based on a relative risk of at least two-fold for women aged 45-70 years, four-fold for women aged 40-44 years and ten-fold for women aged 35-39 years. Details are provided in Appendix 7.1.

Age 45-70 years

1) First degree relative who developed breast cancer at age 50 or less
2) First degree relative who developed bilateral breast cancer
3) Two or more first or second degree relatives who developed breast cancer
4) Nulliparous and a first degree relative who developed breast cancer
5) Benign biopsy with proliferative disease and a first degree relative who developed breast cancer
6) Lobular carcinoma in situ
7) Atypical ductal or lobular hyperplasia in a benign lesion
19) Women at high risk who do not fit into the above categories (risk equivalent)*****

***** These women must have clearly apparent family history indicating at least two fold increased risk of breast cancer.

Age 40-44 years

8) Two or more first or second degree relatives who developed breast cancer at age 50 or less

9) First degree relative with bilateral breast cancer who developed the first breast cancer at age 50 or less

10) Nulliparous and a first degree relative who developed breast cancer at age 40 or less

11) Benign biopsy with proliferative disease and a first degree relative who developed breast cancer at age 40 or less

12) Lobular carcinoma in situ

13) Atypical ductal or lobular hyperplasia in a benign lesion

14) Women at high risk who do not fit into the above categories (risk equivalent)*

***** These women must have clearly apparent family history indicating at least four fold increased risk of breast cancer.

<u>Age 35-39 years</u>

15) Two or more first degree relatives who developed breast cancer at age 50 or less

16) First degree relative with bilateral breast cancer who developed the first breast cancer at age 40 or less

17) Lobular carcinoma in situ

18) Women at high risk who do not fit into the above categories (risk equivalent)*

* These women must have clearly apparent family history indicating at least ten fold increased risk of breast cancer.

NOTE: Approval must first be sought from the Working Party Chairman for all risk equivalents.

If desired an individual clinician may enter only a subset of the eligible women.

2.5 Exclusions

a) Pregnant, or at pregnancy risk. If necessary, pre and peri menopausal women must use <u>non-hormonal</u> contraception during the trial.

b) Any previous cancer (except non-melanoma skin cancer or in situ cancer of the cervix).

c) Life expectancy of less than 10 years or other medical condition more serious than the risk of breast cancer.

d) Psychologically and physically unsuitable for five years tamoxifen/placebo therapy.

e) Current treatment with anti-coagulants.

f) Previous deep vein thrombosis or pulmonary embolus.

g) Current tamoxifen use.

2.6 Hormone Replacement Therapy

Women receiving hormone replacement therapy (HRT) are eligible for the study, and changes in the use of HRT during the study should be noted on the follow-up forms, but this is not grounds for exclusion or withdrawal. Thus, long term use of HRT should be restricted to women for whom a clearly defined medical need exists (e.g. risk factors for cardiovascular diseases or osteoporosis or continuing menopausal symptoms.)

2.7 Randomisation

After completion of the registration form randomisation will be effected by telephone (020 7269 3162) or FAX (020 7269 3429) to the IBIS Co-ordinating Centre.

The next randomised study number for that centre will then be issued which will become the woman's study number and will be used to identify her six-month supply of tablets. All subsequent supplies of tablets will be based on this number. The study will be double blind, i.e. neither the woman nor the doctor will know who is on active treatment.

2.8 Investigations

2.8.1 Mammography

All women will have a physical examination and mammogram (and where necessary fine needle cytology or biopsy) to exclude pre-existing malignancy. A clear mammogram within the twelve months before entry will be accepted. The date and identification number of all mammograms should be recorded to facilitate access to mammograms for central review purposes. During the follow-up period mammography will be performed at intervals, which will be decided locally, or as considered necessary for clinical reasons.

2.8.2 Personal and medical details

A questionnaire giving height, weight, sitting blood pressure, smoking habits, selected medical history and risk factors for breast cancer should be completed for all women entering the study.

Information on prescriptions and hospital visits (other than those directly associated with the study) will be recorded in order to facilitate an assessment of additional costs or savings to the health service related to long term use of tamoxifen. It is difficult to estimate what the outcome of this analysis will be at this stage, since the

costs of diagnosing and treating some side effects (e.g. endometrial or eye problems) may be more than offset by reductions in other areas (e.g. osteoporosis, fractures and heart disease).

2.8.3 Blood samples

Women will have 12 ml venous blood samples taken at entry and 10 ml will be taken at years 1 and 5 as clotted whole blood. 2 ml of the entry sample will be put into the EDTA tube provided and, in the UK, both tubes will be posted immediately by first class postage in pre-addressed containers to Dr Bill Richmond at St. Mary's Hospital, London for determination of total cholesterol, HDL/LDL fractions, triglycerides, apolipoprotein A_1 and apolipoprotein B. Follow-up samples will be treated identically except that the EDTA tube will not be required. Where possible fasting blood samples should be obtained in order that triglycerides can be measured. Bloods will be analysed and results sent back to each centre at regular intervals. Abnormal results will be flagged in accordance with the British Heart Foundation Guidelines and should be sent on to the woman's GP, where appropriate. The follow-up blood samples will also be used to monitor compliance in a sample of women. The blood samples will remain the property of the Working Party, who will be responsible for deciding how they will be used in any further projects.

Arrangements for blood samples taken in other countries will be made in collaboration with the IBIS Co-ordinating Centre on an individual basis, with frozen aliquots being sent to London for long-term storage on a regular basis.

2.8.4 Special Investigations

Measurements of long term changes in hormone levels, detailed lipid profiles, bone densitometry, and endometrial biopsies will be made in selected centres and will be considered as separate studies and are not addressed in this protocol. Data collection will be kept at a minimum and only items that are essential to analyse the specific endpoints listed below will be collected. All protocols for special studies will be submitted to the Working Party for information and co-ordination. A special study on the effects of tamoxifen treatment on psychosocial and sexual functioning organised by Dr. Lesley Fallowfield has already been funded and will be conducted at both Guy's and The Royal Marsden hospitals. It will be important to ensure that special studies do not adversely affect compliance.

2.9 Follow-up

All women will be seen at six-monthly intervals at the hospital clinic during the five years of treatment at which time a short follow-up form will be completed and a supply of tamoxifen or placebo for the next six months will be provided. In exceptional circumstances some of these follow-up visits (but no more than one/year) can be performed by the research nurse over the telephone. Interested GPs, and those within the vicinity of a centre, will be sent the study protocol and information leaflets, as these GPs will be very important both for recruitment

and for support during the study, which should help with compliance. Blood samples to document changes in cholesterol levels and to monitor compliance will be taken at year 1 and year 5. Mammograms will be taken at intervals, to be decided locally. All women will be flagged centrally through the National Registry at Southport, the Scottish Registry or the Northern Ireland Registry for cancer incidence and mortality.

Where necessary hormone replacement therapy may be used to counteract menopausal symptoms. Use should be restricted to that required to alleviate symptoms and the lowest effective dose should be used (e.g. Prempak-C 0.625). Long term use is also allowed where medically indicated. All use of hormone replacement therapy must be fully documented (preparation and duration) in the follow-up forms.

Adherence to treatment protocol will cease if a woman should develop breast cancer (including DCIS). The tamoxifen/placebo code will be broken, and treatment will be according to local practice. Should a woman develop heart disease during the study, it would not be considered necessary for her to leave the study. However, as with any illnesses that develop during the woman's participation in the study, the decision about continuing will rest with the clinician involved.

Should a woman become pregnant during the study, treatment must stop immediately and the code will be broken. If the woman has been receiving the active treatment (tamoxifen) she will be counselled about the possible risks to the fetus and must be referred to an obstetrician for care and advice. For further details see the separate pregnancy protocol (Appendix 7.3).

Monitoring of intake and compliance will be undertaken at regular (six-monthly) intervals.

3 Analysis of Data

3.1 Randomisation

Analysis will be based on randomised treatment option (intent to treat). For this reason it is important to attempt to ensure good compliance before randomisation.

Randomisation will be stratified by individual centre only. The randomisation will not be stratified for the other risk factors, but the factors specified in section 2.4 will be used to achieve retrospective stratification on analysis.

3.2 Endpoints

The primary endpoint is the development of histologically confirmed breast cancer, both invasive and non-invasive (i.e. including DCIS). The principal analysis will be performed when 325 cases of breast cancer have been diagnosed. It is anticipated that this will occur after a median follow-up of 7 years.
Adherence to treatment protocol will cease if a woman develops breast cancer and a breast cancer report form should be returned. All breast cancer pathology must be reviewed by a pathologist approved by the National Breast Screening Programme. It is recognised that breast cancer mortality is a very important endpoint and this will also be analysed. However, the power to detect this within 10 years is marginal and an overview of all world wide trials of tamoxifen may be needed to get clear results on this question. Total and cause-specific mortality will be analysed and the final report form should be returned for any woman who dies of any cause while on therapy. All women will be flagged for cancer incidence and cause of death. An important secondary endpoint will be the occurrence of and mortality from myocardial infarction, which could be significantly reduced. To facilitate this comparison height, weight, smoking history, seated blood pressure and cholesterol levels will be recorded at entry. Blood pressure will also be recorded on follow-up visits and blood will be taken for cholesterol assays at years 1 and 5. During the period of active follow-up (5 years) other serious medical conditions will also be recorded including myocardial infarction, thromboembolic events, other cardiovascular events, fractures, other cancers and eye problems. Basic information on prescriptions and hospital visits will also be recorded to facilitate an overall cost-benefit analysis of tamoxifen usage.

3.3 Numbers of Volunteers and Power

Three factors determine the numbers needed for this study. They are (i) the absolute risk level of the participants, (ii) the size of the smallest reduction thought to be worthwhile and (iii) compliance rates. Assuming a baseline risk of 1.6 cases of breast cancer per 1,000 women per year and a 2½ fold overall average relative risk, eligible women would have a rate of breast cancer of 4 cases per 1000 women-years. Assuming a 40% reduction of breast cancer in the treated arm after 5 years of treatment, a 5% significance level and a 75% average compliance, the power to detect such a change with 3500 women in each arm is 78%, after 10 years of follow-up. If the reduction of breast cancer is one half this will be detected in 7 years with 83% power. Details are given in Appendix 7.2. Smaller differences and results in subgroups will be detectable as part of an overview with other studies. The vast majority of women are expected to come from the 45-70 year age group. This will be monitored during the study and sample size calculations will be reassessed if necessary.

3.4 Data Monitoring

Interim analysis of the data will be undertaken at 6 monthly intervals and provided to the Data Monitoring Committee. The Data Monitoring Committee will have the responsibility to recommend whether to stop the study early. All serious/life threatening possible adverse events should, in addition to the study documentation, be reported to the IBIS Co-ordinating Centre immediately, and subsequently to the Data Monitoring Committee. As a general guideline, a difference between arms should exceed three standard deviations for some mortality endpoint before a decision to stop prematurely is taken, although other factors will also have to be taken into consideration.

4 Toxicity and Hazards

Tamoxifen is a comparatively well tolerated drug with minimal reported side effects. In a placebo controlled trial in well women (Powles *et al.*, 1990), it was found that non-specific side effects such as nausea, vomiting and headaches were no more common for tamoxifen than for the placebo group. Menopausal hot flushes, vaginal discharges and vaginal dryness were slightly more common for tamoxifen than for placebo but these were minor, and did not materially affect compliance. Intolerance occurs rarely and the relevant side effects usually resolve rapidly on cessation of medication. Side effects which have been reported to the manufacturer include light-headedness, skin rash, fluid retention, thrombocytopaenia, gastrointestinal disturbances, alopecia, one report of a change to the singing voice (Goodare, 1992), visual problems (Kaiser-Kupfer *et al.*, 1978; Bentley *et al.*, 1992), vaginal bleeding and in premenopausal women cystic ovarian swellings and suppression of menstruation.

Little evidence exists on the teratogenic effects of tamoxifen in humans, but as a precaution pregnant women, those wishing to have children or who are considered to be at risk of pregnancy should be excluded. Should a woman become pregnant during the study, treatment will cease and the code will be broken (see section 2.8 and Appendix 7.3).

A compilation of all new primary tumours in sites other than the breast among 14,000 patients in randomised trials has yielded the same incidence among the women treated with tamoxifen as for controls (Jackson *et al.*, 1991). However, the Swedish group (Fornander *et al.*, 1989) found an excess of endometrial tumours in their trial, using a dose twice that to be used in this study (40 mg/day vs. 20 mg/day). Excesses have been reported in some trials using 20 mg/day (Sunderland and Osborne, 1991; Andersson *et al.*, 1992; NSABP, 1992) but not in others (NATO, 1988 (Nayfield *et al.*, 1991); Ribeiro and Swindell, 1991; ECOG (Nayfield *et al.*, 1991); Stewart, 1992). No tumours except endometrial cancer were found in excess in a large cohort study (Curtis *et al.*, 1996) .Although these tumours generally have a good prognosis, prompt investigation of any irregular

bleeding or other symptoms of endometrial cancer is advised. Other endometrial abnormalities that have been observed in tamoxifen treated patients include endometrial hyperplasia (Buckley, 1990) and endometrial polyps (Neven *et al.*, 1989).

Only 7 cases of liver cancer among patients in trials of tamoxifen have been reported, 4 in patients receiving tamoxifen and 3 in controls not receiving tamoxifen (Rutqvist *et al.*, 1995). No cases have been reported in the Scottish trial in which 1,323 women were randomised to receive 20mg tamoxifen per day for 5 years (as in this trial) and who have been followed for more than 10 years (Stewart *et al.*, 1989) or a similar American trial (Fisher *et al.*, 1994). Only one other case has been reported in the literature. Uncertainty remains about the risk after ten years, and it is possible that some cases have not been reported because of confusion with the spread of breast cancer to the liver but, overall, the available evidence for a possible increase in liver cancer from studies in women is reassuring. In particular no excess has been found in a large survey of breast cancer patients followed up since 1977, when tamoxifen was introduced in the United States (Muhlemann *et al.*, 1994).

Two anecdotal reports of liver damage in patients taking tamoxifen have appeared (Blackburn *et al.*, 1984; Ching *et al.*, 1992) but in neither case could the damage clearly be seen to be due to tamoxifen usage. A few further cases of liver toxicity have been reported to the authorities but, again, whether they were caused by tamoxifen is unclear.

High doses of tamoxifen have been shown to produce liver tumours in rats, but not in mice (Greaves *et al.*, 1993). Nevertheless, in this study rats on the lowest dose (which is still more than ten times higher than the human dose) lived longer than control rats not receiving tamoxifen (Greaves *et al.*, 1993).

Changes in the DNA (DNA adducts) have also been reported in the liver of rats treated with tamoxifen (Han *et al.*, 1992; White *et al.*, 1992), and to a lesser extent in mice (White *et al.*, 1992). However, the relevance of these observations to women is uncertain, and one small study has not found adducts in the liver of women receiving tamoxifen (Martin *et al.*, 1995). Another study has not found adducts in the endometrium of women (Carmicheal *et al.*, 1996).

Thromboembolic complications have been reported in patients receiving both tamoxifen and chemotherapy (Lipton *et al.*, 1984), but these seem most often to be associated with use of the treatments in combination (Saphner *et al.*, 1991). An excess of thromboembolic disease was seen in two studies using tamoxifen alone (Stewart *et al.*, 1989; McDonald, 1995), but not in another one (Rutqvist *et al.*, 1993). Antithrombin III (a substance which prevents blood clots) is slightly depressed by tamoxifen use, but this does not appear to affect the well-being of the individual (Jordan *et al.*, 1989).

NOTE Tamoxifen affects the action of treatment given to prevent blood clots eg. WARFARIN. Therefore the two drugs should not be taken together in the study.

4.1 <u>Unblinding the Randomisation</u>

The study is a double blind, placebo controlled, randomised trial i.e. neither the doctor nor the woman will know who is on active treatment.

The tamoxifen/placebo code should only be broken in the following circumstances:

1) The woman develops breast cancer
2) The woman becomes pregnant
3) Where it is considered necessary by any clinician involved in her care.

The necessity for verbal code breaking is highly unlikely and therefore the usual procedure would be a written request with precise details of the situation.

In exceptional circumstances the code can be broken by telephoning the Senior Study Co-ordinator (020 7269 3151) or IBIS Chairman, Dr Jack Cuzick (020 7269 3006) during working hours. Outside these hours telephone 020 7242 0200 and an emergency number will be given.

4.2 <u>Adverse Events</u>

All local centres must inform the IBIS Co-ordinating Centre as soon as possible of all events which <u>are both</u>:

i) probably or definitely related to study therapy

and

ii) serious (fatal, life threatening, leading to hospitalisation or prolongation of hospital stay or leading to persistence of disability, or incapacity).

The IBIS Co-ordinating Centre will notify the Committee on Safety of Medicines (CSM) of all adverse events. Clinicians should inform the IBIS Co-ordinating Centre if they have informed the CSM directly.

5 Consent and Ethics Committee Approval

The feasibility study has been approved by the Royal Marsden Hospital Ethics Committee. The main study will require approval by the ethics committees of participating centres, who must receive a copy of the Department of Health's letter of June 10th 1993 setting out the basis by which the study may be run within the NHS.

This study involves evaluation of tamoxifen for an unlicensed indication and is being carried out under CTC no. 11962/0001. The use of tamoxifen in this study is a new indication of a licensed drug which is already widely used in clinical practice. Responsibility for its use in an individual woman lies with the doctor involved and his/her local district health authority. No special indemnity is required and details of compensation for any injury will follow the Department of Health's guidelines for Local Research Ethics Committees.

All women interested in participating in the study will receive an information leaflet which explains the purpose of the study and warns of the potential short term and long term side effects of tamoxifen. They will also be given a leaflet detailing what taking part in the study entails (Appendix 7.6). For those women who would like further, more scientific material, a detailed information document will be available (Appendix 7.7). The clinician will clarify and discuss any points arising from the information literature and participants will then be required to sign an informed consent form (Appendix 7.8). Further information and counselling will be made available throughout the study in response to a woman's request or following significant new information on the side effects of tamoxifen. This will be provided by the clinician or the research nurse appointed for this study. All women will be free to stop treatment at any stage, and it will be made clear that leaving the study will not prejudice any future treatment which may be needed.

6 References

Andersson, M., Storm, H.H. & Mouridsen, H.T. (1992) Carcinogenic effects of adjuvant tamoxifen treatment and radiotherapy for early breast cancer.
Acta Oncol.; **31**:259-263.

Bentley, C.R., Davies, G. & Aclimandos, W.A. (1992) Tamoxifen retinopathy: a rare but serious complication.
BMJ; **304**:495-496.

Blackburn, A., Amiel, S., Millis, R. *et al.* (1984) Tamoxifen and liver damage.
BMJ; **289**:288.

Bruning, P.F., Bonfrer, J.M.G., Hart, A.A.M. *et al.* (1988) Tamoxifen, serum lipoproteins and cardiovascular risk. *Br. J. Cancer*; **58**:497-499.

Buckley, C.H. (1990) Tamoxifen and endometriosis. Case report.
B. J. Obs. & Gynae.; **97**:645-646.

Caleffi, M., Fentiman, I.S., Clark, G. *et al.* (1988) Effect of tamoxifen on oestrogen binding lipid and lipoprotein concentration and blood clotting parameters in premenopausal women with breast pain.
J. Endocrinol.; **119**:335-339.

Ching, C.K., Smith, P.G. & Long, R.G. (1992) Tamoxifen-associated hepatocellular damage and agranulocytosis. *Lancet*; **339**:940.

Cuzick, J. & Baum, M. (1985) Tamoxifen and contralateral breast cancer.
Lancet; **i**:282.

Cuzick, J., Wang, D.Y. & Bulbrook, R.D. (1986) The prevention of breast cancer.
Lancet; **i**:83-86.

Dupont, W.D. & Page, D.L. (1985) Risk factors for breast cancer in women with proliferative breast disease.
N. Engl. J. Med.; **312**:146-151.

EBCTCG (1992) Systemic treatment of early breast cancer by hormonal, cytotoxic or immune therapy.
Lancet; **339**:1-15 / 71-85.

Fentiman, I.S. (1989) The endocrine prevention of breast cancer.
Br. J. Cancer; **60**:12-14.

Fentiman, I.S., Caleffi, M., Rodin, A. *et al.* (1988) Bone mineral content of women receiving tamoxifen for mastalgia. *Br. J. Cancer*; **60**:262-264.

Fentiman, I.S. & Powles, T.J. (1987) Tamoxifen and benign breast problems.
Lancet, **ii**:1070.

Fisher, B., Costantino, J., Redmond, C. *et al.* (1989) A randomised clinical trial evaluating tamoxifen in the treatment of patients with node negative breast cancer who have oestrogen receptor positive tumors.
N. Engl. J. Med.; **320**:479-484.

Fornander, T., Cedermark, B., Mattsson, A. *et al.* (1989) Adjuvant tamoxifen in early breast cancer: occurrence of new primary cancers. *Lancet*; **i**:117-120.

Goodare, H. (1992) Adjuvant treatment in breast cancer. (letter) *Lancet*; **339**:424.

Greaves, P., Goonetilleke, R., Nunn, G. *et al.* (1993) Two year carcinogenicity study of tamoxifen in Alderley Park Wistar-derived rats. *Cancer Res.*; **53**:3919-3924.

Han, X. & Liehr, J.G. (1992) Induction of covalent DNA adducts in rodents by tamoxifen.
Cancer Res.; **52**:1360-1363.

Hirayama, T. & Wynder, E.L. (1962) Study of epidemiology of cancer of the breast: influence of hysterectomy.
Cancer; **15**:28-38.

Jackson, I.M., Litherland, S. & Wakeling, A.E. (1991) Tamoxifen and other antioestrogens.
In *Medical Management of Breast Cance*r, ed: Powles, T.J. & Smith, I.E. Publ. Martin Dunitz, London.

Jordan, V.C. (1981) Comparative anti-oestrogen action in experimental breast cancer.
Adv. Exp. Med. Biol.; **138**:165.

Jordan, V.C., Naylor, K.E., Dix, C.J. *et al.* (1980) Anti-oestrogen action in experimental breast cancer. *Recent Results Cancer Res.*; **10**:34.

Kaiser-Kupfer, M.I. & Lippman, M.E. (1978) Tamoxifen retinopathy.
Cancer Treat. Rep.; **62**:315-320.

Kelsey, J.L. (1979) A review of the epidemiology of human breast cancer.
Epidemiol. Revs.; **1**:74-109.

Lipton, A., Harvey, H. & Hamilton, R. (1984) Venous thrombosis as a side-effect of tamoxifen treatment.
Cancer Treat. Rep.; **68**:887-889.

Love, R.R., Newcomb, P.A., Wiebe, D.A. *et al.* (1990) Effects of tamoxifen therapy on lipid and lipoprotein levels in postmenopausal patients with node-negative breast cancer.
J. Natl. Cancer Inst.; **82**:1327-1332.

MacMahon, B. & Feinlieb, M. (1960) Breast cancer in relation to nursing and menopausal history.
J. Natl. Cancer Inst.; **24**:733-753.

McDonald, C.C. & Stewart, H.J. for the Scottish Breast Cancer Committee (1991) Fatal myocardial infarction in the Scottish adjuvant tamoxifen trial.
BMJ; **303**:435-437.

Miller, A.B. & Bulbrook, R.D. (1980) The epidemiology and etiology of breast cancer.
N. Engl. J. Med.; **303**:1246.

NATO (1988) Controlled trial of tamoxifen as a single adjuvant agent in the management of early breast cancer: Analysis at eight years by Nolvadex Adjuvant Trial Organisation.
Br. J. Cancer; **57**:608-611.

Nayfield, S.G., Karp, J.E., Ford L.G. *et al.* (1991) Potential role of tamoxifen in the prevention of breast cancer. *J. Natl. Cancer Inst.*; **83**:1450-1459.

Neven, P., De Muylder, X., Van Belle, Y. *et al.* (1989) Tamoxifen and the uterus and endometrium.
Lancet; **21**:375.

NSABP (1992) Breast cancer prevention trial protocol; P-1.

Powles, T.J., Hardy, J.R., Ashley, S.E. *et al.* (1989) A pilot trial to evaluate the acute toxicity and feasibility of tamoxifen for prevention of breast cancer.
Br. J. Cancer; **60**:126-131.

Powles, T.J., Tillyer, C.R., Jones, A.L. *et al.* (1990) Prevention of breast cancer with tamoxifen - an update on the Royal Marsden Hospital pilot programme.
Eur. J. Cancer; **(26)6**:680-684.

Ribeiro, G. & Swindell, R. (1991) The Christie Hospital adjuvant tamoxifen (Nolvadex) breast trial: an analysis at a maximum follow-up of 13 years. In Current Controversies in the Treatment of Breast Cancer, Volume 2, The Nottingham Proceedings. Published by Parthenon.

Rossner, S. & Wallgren, A. (1984) Serum lipoprotein after breast cancer surgery and effects of tamoxifen.
Atherosclerosis; **52**:339.

Rutqvist, L.E. & Mattson, A. (1993) Cardiac and thromboembolic morbidity among postmenopausal women with early-stage breast cancer in a randomised trial of adjuvant tamoxifen.
J. Natl. Cancer Inst.; **85**: 1398-1406.

Saphner, T., Tormey, D.C. & Gray, R. (1991) Venous and arterial thrombosis in patients who received adjuvant therapy for breast cancer.
J. Clin. Oncol.; **9(2)**:286-294.

Stewart, H.J. & Knight, G.M. (1989) Tamoxifen and the uterus and endometrium.
Lancet; **i**:375.

Stewart, H.J. for the Scottish Cancer Trials Breast Group (1992) The Scottish trial of adjuvant tamoxifen in node-negative breast cancer.
J. Natl. Cancer Inst. Monogr.; **11**:117-120.

Sunderland, M.C. & Osborne, C.K. (1991) Tamoxifen in premenopausal patients with metastatic breast cancer: a review.
J. Clin. Oncol.; **9**:1283-1297.

Turken, S., Siris, E., Seldin, D. *et al.* (1989) Effects of tamoxifen on spinal bone density in women with breast cancer.
J. Natl. Cancer Inst.; **81**:1086-1088.

White INH, de Matteis F, Davies A et al.: Genotoxic potential of tamoxifen and analogues in female Fischer F344/n rats, DBS/2 and C57B1/6 mice and in human MCL-5 cells.
Carcinogenesis; **13**:2197-2203,1992.

Neal AJ, Evans K & Hoskin PJ: Does long-term administration of tamoxifen affect bone mineral density?
Eur J Cancer **29A**:1971-1973, 1993.

Rutqvist LE, Johansson H: Adjuvant Tamoxifen Therapy for Early Stage Breast Cancer and Second Primary Malignancies.
J Natl Cancer Inst **87**: 645-651, 1995.

Greaves P, Goonetilleke R, Nunn G *et al*: Two-year carcinogenicity study of tamoxifen in Alderley Park wistar-derived rats.
Cancer Res **53**: 3919-3924, 1993.

Fisher B, Costantino J P, Richmond C K, *et al* Endometrial cancer in tamoxifen-treated breast cancer patients: Findings from the National Surgical Adjuvant Breast and Bowel Projects (NSABP) B-14.
J Natl Cancer Inst **86**: 527-537, 1994

Powles J T, Jones L A , Ashley E S *et al*: The Royal Marsden Hospital pilot tamoxifen chemoprevention trial.
Br Cancer Research & Treatment **31**: 73-82, 1994.

Muhlemann K, Cook S L & Weiss S W: The incidence of hepatocellular carcinoma in US white women with breast cancer after the introduction of tamoxifen in 1977.
Br Cancer Research & Treatment **30**: 201-204, 1994.

Zhang J J, Jacob T J C, Velverde M A *et al*: Tamoxifen Blocks Chloride Channels: A Possible Mechanism for Cataract Formation.
J Clin. Invest. **94(10)**: 1690-1697, 1994.

Neven P, De Muylder X, Van Belle Y *et al*: Tamoxifen and the uterus. British Med J 309: 1313-1314, 1994. and Bissett D, Davis JA & WD George: Gynaecological monitoring during tamoxifen therapy.
Lancet **344** : 1244, 1994.

Kedar RP, Bourne HT, Powles T *et al*: Effects of tamoxifen on uterus and ovaries of postmenopausal women in a randomised breast cancer prevention trial.
Lancet **34**3: 1318-21, 1994.

Evans MJ, Langlois NEI, Kitchener HC et al: Is there an association between long-term tamoxifen treatment and the development of carcinosarcoma (malignant mixed Mullerian tumor) of the uterus?.
Int J Gynecol Cancer **5**: 310-313, 1995.

McDonald C C, Alexander F E, Whyte B W et al: Cardiac and vascular morbidity in women receiving adjuvant tamoxifen for breast cancer in a randomised trial.
BMJ **311**: 977-980, 1995.

Martin E A, Rich K J, White I N H et al: ^{32}P-Postlabelled DNA adducts in liver obtained from women treated with tamoxifen.
Carcinogenesis **16(7)**: 1651-1654, 1995.

Powles T J , Hickish T, Kanis J A et al: The Effect of Tamoxifen on Bone Mineral Density Measured by Dual Energy Xray Absorptiometry in Healthy Pre and Postmenopausal Women.
J Clinical Oncology **14**: 78-84, 1996.

Cook L S, Weiss N S & Schwartz S M et al: Population-Based Study Tamoxifen Therapy and Subsequent Ovarian, Endometrial, and Breast Cancer.
J Natl Cancer Inst **87**: 1359-1364, 1995.

Sasco A J, Chaplain G & Amoros E et al: Endometrial Cancer following Breast Cancer: Effect of Tamoxifen and Castration by Radiotherapy.
Epidemiology **7**: 9-13, 1996.

Carmicheal P L, Ugwumadu A H N & Neven P et al: Lack of Genotoxicity of Tamoxifen in Human Endometrium.
Cancer research **56**: 1475-1479, 1996.

Assikis V J & Jordan V C: Gynecologic effects tamoxifen and the association with endometrial carcinoma.
Intl J Gynae Obstet **49(3)**: 241-257, 1995.

Neven P: Endometrial changes in patients on tamoxifen.
Lancet **346**:1292, 1995.

Kiristensen B, Ejlertsen B, Henning T et al: Femoral fractures in postmenopausal breast cancer patients treated with adjuvant tamoxifen.
Br Cancer Res Treat **39**: 321-326, 1996.

Early Breast Cancer Trialists' Collaborative Group: Tamoxifen for early breast cancer: an overview of the randomised trials.
Lancet **351**: 1451-1467, 1998.

Fisher B, Costantino J P, Wickerham D L, et al: Tamoxifen for Prevention of Breast Cancer: Report of the National Surgical Adjuvant Breast and Bowel Project P-1 Study.
Journal of the National Cancer Institute **90(18)**: 1371-1387, 1998.

Veronesi U, Maisonneuve P, Costa A, et al: Prevention in breast cancer with tamoxifen: preliminary findings from the Italian randomised trial among hysterectomised women.
Lancet **352**: 93-97, 1998.

Powles T, Eeles R, Ashley S, et al: Interim analysis of the incidence of breast cancer in the Royal Marsden Hospital tamoxifen randomised chemoprevention trial.
Lancet **352**: 98-101, 1998.

7.1 Entry criteria: International Breast Cancer Intervention Study

AGE 45-70: INCREASED RISK GROUP - Relative risk of breast cancer at least 2-fold (corresponds to life-time risk of 15% or 1 in 7).

Criterion	Estimated relative risk	Approximate number of new women eligible each year
First degree relative who developed BC at age 50 or less	~ 2	5000 x 0.8 (av. number daughters and sisters of proband) = 4000
First degree relative who developed bilateral BC	~ 3	4% x 25000 x 0.8 = 800
Two or more first or second degree relatives who developed BC	> 2	10% x 10% = 1% of population = 2000
Nulliparous and a first degree relative who developed BC	1.5 x 1.5 = 2.2	15% x 5% = 0.75% of population = 1500
Benign biopsy with proliferative disease* and a first degree relative who developed BC	1.6 x 1.5 = 2.4	0.8 x 25000 x 3% = 600
Lobular carcinoma in situ (LCIS)	~ 10	2.5% of screen detected BC<br>$(1.0 \times 10^{6}) \times (2 \times 10^{-3}) \times (0.025) = 50$
Atypical ductal or lobular hyperplasia in a benign lesion†	4	4% of benign lesions ≅ 600

AGED 40-44: HIGH RISK GROUP - Relative risk at least 4-fold

Two or more first or second degree relatives who developed BC at age 50 or less	~ 5	5% x 4000 = 200
First degree relative with bilateral BC who developed the first BC at age 50 or less	~ 5	5000 x 0.5 x 4% x 0.8 = 80
Nulliparous and first degree relative who developed BC at age 40 or less	1.5 x 2.7 = 4	15% x 0.8 x 1400 = 168
Benign biopsy with proliferative disease and a first degree relative who developed BC at age 40 or less	1.6 x 2.7 = 4.3	0.8 x 1400 x 3% = 30
LCIS	~ 10	5% of invasive rate<br>1500 x 0.05 = 75
Atypical ductal or lobular hyperplasia in a benign lesion†	4	~ 300

AGE 35-39: VERY HIGH RISK GROUP - Relative risk at least 10-fold

Two or more first degree relatives who developed BC at age 50 or less	~ 10	5% of 4000 = 200
First degree relative who developed bilateral BC at age 40 or less	~ 10	1/3 of cases at age 40 or less<br>0.8 x 1/3 x 1400 ≅ 370
LCIS	~ 10	5% of invasive rate<br>1400 x 0.05 = 70

These are defined in 'Pathology Reporting in Breast Cancer Screening', pages 19-21:-

† Atypical ductal or lobular hyperplasia - 'possessing some but not all of the characteristic features of ductal or lobular carcinoma in situ' (Dupont & Page, 1985).

* 'Epithelial proliferation without atypia - This term is used to describe all cases of intra-luminal proliferation lacking atypia where the cells are more than 4 thick. The change may involve terminal duct lobular units or inter-lobular ducts.'

7.2 Revised Power Calculations (01/10/96)

	5 yrs	7 yrs	7 yrs	10 yrs
Baseline risk	1.6×10^{-3} cases/ yr			
Relative risk	2.5			
Cases/1000	20	28	28	40
Cases in 3500 women	70	98	98	140
	**50% reduction**		**40% reduction**	
Cases in breast group (0)	35	49	58.8	84
Expected cases in treatment group	52.5	73.5	78.4	112
$Z^2 = (O\text{-}E)^2 / (^1/_2E)$	11.67	16.33	9.8	14
Reduction for compliance $\gamma^2(1-{}^1/_2\Delta) / (1-{}^1/_2\Delta\delta)$ $\gamma = 0.75$	6.06	8.48	5.19	7.41
$Z_{\alpha/2} + Z_\beta$	2.46	2.91	2.28	2.72
Z_β	0.50	0.95	0.31	0.76
β (power)	69%	83%	62%	78%

7.3 Recruitment Update

After the DMC meeting of October 1996, it was concluded that a further 3 years recruitment of 150/month would produce a trial of about 7000 women. Revised power calculations indicate that a trial of this size would have 86% power to detect a 50% reduction in breast cancer incidence after 7 years of follow up and power of 80% for a 40% reduction after 10 years. These reductions are in line with the newer data from the Oxford overview. Additional power to detect smaller differences or to look at subgroups of patients will require combining results with the other prevention study. Agreement to do this was established at the outset for the Royal Marsden pilot trial (2500 women) and the Italian trial (current entry 5000, expected entry 8000-10,000). Agreement to combine results with the American trial has also been obtained on an informal basis, but they will wish to maintain greater independence until publication.

The importance of maintaining our contribution to an overview is underscored by the difficulties in recruitment worldwide. The American trial has decided to limit entry to 13,000 because their risk profile was substantially greater than initially designed for, and recent recruitment has been slow. The Italian trial is also unlikely to meet its original target (25,000), so an overview is likely to be necessary to obtain useful results in this important but difficult area.

7.4 Pregnancy Protocol

INTERNATIONAL BREAST CANCER INTERVENTION STUDY

Pregnancy risk procedure

Introduction

No evidence for a teratogenic effect of tamoxifen has been found in animal studies. However, there are no adequate and well controlled studies in pregnant women, so doubt exists. The drug is contra-indicated in pregnant women. Tamoxifen is used in the treatment of anovular fertility.

For these reasons we advise the following procedures:

1 Contraception

Eligible

a) Sterilised
b) Partner sterilised
c) No partner
d) Effective barrier contraceptive/IUD

Not eligible

a) Oral contraceptive
b) Other unreliable methods of contraception
c) Contemplating further pregnancies in the future

2 Changed circumstances

The no risk of pregnancy status at the time of inclusion into the study and start of tamoxifen/placebo may change (such as a new partner) to a significant risk status or even planned pregnancy status. Participants in these latter two categories should stop tamoxifen/placebo immediately.

3 Risk of pregnancy

Some women will wish to use barrier contraception or an IUD. They are still eligible to be included in the study in spite of an uncertain risk of pregnancy. However, before entering the study the potential risks associated with pregnancy must be fully explained and attention drawn to the relevant paragraphs in the consent form.

4 Pregnancy

Should any compliant participant become pregnant:

a) medication must stop immediately
b) code must be broken

If tamoxifen:

c) risks of coincidental fetal abnormality and unknown teratogenic risk discussed and witnessed
d) participants must be referred to an independent obstetrician for care and advice on further action including:

    (i) Continuing the pregnancy
    (ii) Further investigations.
    (iii) Termination of pregnancy.
